# Molecular determinants of TRAF6 binding specificity suggest that native interaction partners are not optimized for affinity

**DOI:** 10.1002/pro.4429

**Published:** 2022-10-26

**Authors:** Jackson C. Halpin, Dustin Whitney, Federica Rigoldi, Venkat Sivaraman, Avinoam Singer, Amy E. Keating

**Affiliations:** ^1^ MIT Department of Biology Cambridge Massachusetts USA; ^2^ MIT Department of Biological Engineering Cambridge Massachusetts USA; ^3^ Koch Institute for Integrative Cancer Research Cambridge Massachusetts USA

**Keywords:** binding specificity, inhibitor, molecular modeling, peptide library, short linear motif, TRAF6

## Abstract

TRAF6 is an adaptor protein involved in signaling pathways that are essential for development and the immune system. It participates in many protein–protein interactions, some of which are mediated by the C‐terminal MATH domain, which binds to short peptide segments containing the motif PxExx[FYWHDE], where x is any amino acid. Blocking MATH domain interactions is associated with favorable effects in various disease models. To better define TRAF6 MATH domain binding preferences, we screened a combinatorial library using bacterial cell‐surface peptide display. We identified 236 of the best TRAF6‐interacting peptides and a set of 1,200 peptides that match the sequence PxE but do not bind TRAF6 MATH. The peptides that were most enriched in the screen bound TRAF6 tighter than previously measured native peptides. To better understand the structural basis for TRAF6 interaction preferences, we built all‐atom structural models of the MATH domain in complex with high‐affinity binders and nonbinders identified in the screen. We identified favorable interactions for motif features in binders as well as negative design elements distributed across the motif that can disfavor or preclude binding. Searching the human proteome revealed that the most biologically relevant TRAF6 motif matches occupy a different sequence space from the best hits discovered in combinatorial library screening, suggesting that native interactions are not optimized for affinity. Our experimentally determined binding preferences and structural models support the design of peptide‐based interaction inhibitors with higher affinities than endogenous TRAF6 ligands.

## 1 INTRODUCTION

Protein–protein interactions assemble signal transduction networks that are critical for cellular function and are often implicated in disease. Knowledge of which proteins interact, and how, is essential for a mechanistic understanding of information propagation in cells and how these networks are perturbed in disease. Identifying molecules that can mimic and compete with native binding partners provides compounds that can be used as research tools; such inhibitors may also have potential to be developed as therapeutics.

Myriad interactions important for signaling involve the binding of a recognition domain in one protein by a short linear interaction motif (SLiM) in a partner protein. Many such domain/motif pairs, including the TRAF6 MATH domain/TRAF6‐interaction motif pair that is the subject of this work, have been compiled in the Eukaryotic Linear Motif database.^1^ Most motif definitions are based on patterns found in a few examples, leading to incomplete models that do not fully capture the sequence features necessary or sufficient for binding in the cell. A deeper understanding of SLiM sequence requirements can come from large‐scale screens, which can provide a more comprehensive view of protein recognition domain specificity.^2^, ^3^, ^4^, ^5^, ^6^, ^7^

TRAF6 is a member of the tumor necrosis factor receptor‐associated factor (TRAF) family of adaptor proteins with E3 ubiquitin ligase functions.^8^, ^9^, ^10^ TRAF6 mediates NF‐κB signaling and thereby participates in immunity and inflammation‐related pathways. TRAF6 binds directly or indirectly to tumor necrosis factor receptors and members of the interleukin‐1 (IL‐1) receptor/Toll‐like receptor superfamily, among other proteins. Downstream targets for TRAF6‐mediated K63‐linked ubiquitylation connect to the regulation of proteins such as transforming growth factor‐β‐activated kinase‐1 (TAK1), IκB kinase (IKK), and mitogen‐activated protein (MAP) kinases, which subsequently lead to the regulation of NF‐κB and AP‐1 activity.^9^, ^11^ Direct inhibition of the C‐terminal domain of TRAF6 (the TRAF‐C Meprin and TRAF Homology—or MATH—domain) has been proposed and explored as a potential therapeutic strategy for the treatment of a variety of pathologies such as cardiovascular diseases, diseases associated with obesity, osteoporosis, and others.^12^, ^13^, ^14^, ^15^, ^16^, ^17^, ^18^

**FIGURE 1:**
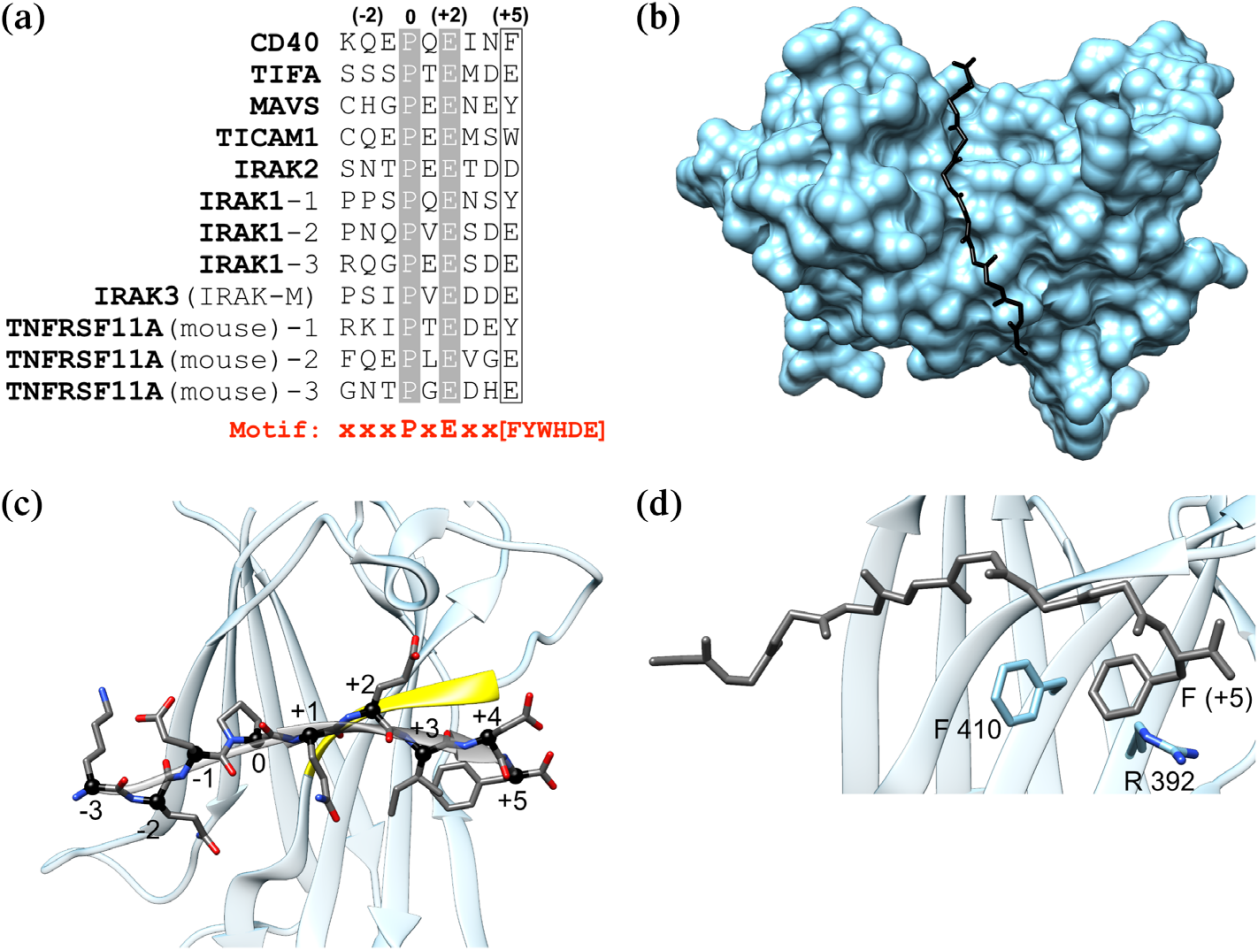
TRAF6 MATH domain interactions with TIM6 peptide ligands. (a) Alignment of TRAF6‐binding sequences from known partners showing the numbering scheme used throughout this paper. (b–d) Structure of the TRAF6 MATH domain (cyan) bound to the CD40* peptide (dark grey), which includes a point mutation relative to human CD40, PDB ID 1LB6. (b) MATH domain in surface representation bound to the CD40* peptide. (c) Bound peptide with positions numbered as in (a). Peptide residues at (+1)–(+5) form a beta‐strand that pairs with the MATH domain (paired strand in yellow). (d) Interaction of the (+5) Phe in CD40* with Phe 410 and Arg 392 in TRAF6.

TRAF6, like other members of the TRAF family, has four domains. The N‐terminal RING domain works with the zinc finger domains as an E3 ubiquitin ligase. A coiled‐coil domain trimerizes TRAF6. The 17.4 kDa C‐terminal MATH domain engages peptides containing TRAF interaction motifs (TIMs) and is responsible for cellular localization.^19^ The MATH domains of TRAFs 1, 2, 3, and 5 share high sequence similarity, whereas TRAF4 and TRAF6 are more diverged in sequence and function.^9^, ^20^, ^21^, ^22^ TRAF6 MATH is reported to bind peptides that contain the motif xxxPxExx[FYWHDE] (here referred to as TIM6; Figure 1a), where x is any amino acid. TRAFs 1, 2, 3, and 5 have been reported to bind [PSAT]x[QE]E, PxQxxD, and PxQxT motifs,^1^ although other work suggests deviations from these definitions.^28^ For TRAF6, the proline and glutamate residues, referenced here as motif positions (0) and (+2), appear strictly conserved for TRAF6 binding.^19^, ^23^, ^24^, ^25^, ^26^, ^27^, ^28^ A preference for aromatic or acidic residues at (+5) is maintained in peptides that have been experimentally validated to bind to the TRAF6 MATH domain (Figure 1a).^19^, ^23^, ^24^, ^25^, ^26^, ^27^, ^28^ Structures show that the TRAF6 MATH domain binds to peptides that extend a beta‐sheet in the MATH domain (Figure 1b–d).^19^, ^23^, ^24^ Residues in position (+5) bind in a pocket comprised of aromatic and basic residues, engaging in electrostatic and pi‐pi interactions (Figure 1d).

Given the low complexity of the TRAF6 motif PxExx [FYWHDE], we reasoned that there might be other determinants of high‐affinity TRAF6 binding. To define motif‐proximal features important for the interaction of SLiMs with the TRAF6 MATH domain, we used bacterial surface‐display screening to explore sequence preferences within a library denoted xxxPxExxx, with x being a random amino acid, keeping the proline fixed at position (+0) and the glutamate fixed at position (+2). We screened this library and identified 236 highly enriched binders and 1,200 nonbinders. We then used structure‐based modeling to examine the interaction between the peptides and the MATH domain. Our analysis revealed residues within the motif that support high‐affinity binding and negative‐design elements that explain why many peptides that contain PxE are not suitable TRAF6 ligands. These insights help to elucidate the determinants of TRAF6 binding affinity. We compared the sequence features of the library‐identified binders with reported native TRAF6 binders and found that most native interaction partners do not match the top sequences isolated from the library. Notably, there are no sequences in the human proteome that share all of the features that are prominent among the tightest binders from the screen. These results suggest that native TRAF6 interaction partners may be under functional selection for moderate affinity, and is consistent with observations that other factors, such as ligand oligomerization, are important for triggering TRAF6 binding in certain biological contexts.^9^, ^29^ The lack of high‐affinity binders in the proteome provides an opportunity to out‐compete native interactions using designed peptides or mini‐proteins that have features uncovered in our screen.

## 2 RESULTS

### 2.1 Library screening by cell‐surface display reveals strong positional preferences for peptides that bind TRAF6

Bacterial‐surface display can provide information about the binding of short peptides to protein interaction domains.^3^, ^30^ For this work, we developed surface‐display constructs in which TIM6 peptides were fused to the C‐terminus of re‐engineered OmpX,^31^, ^32^ such that when the construct was expressed, the TIM6 peptides were presented on the outer membrane of *Escherichia coli* cells. To measure binding to TRAF6, peptide‐displaying cells were incubated with biotinylated TRAF6 homotrimers consisting of the coiled‐coil and MATH domains (construct termed T6cc). The amount of TRAF6 bound to the cells was then quantified by adding streptavidin‐conjugated phycoerythrin and analyzing the cells via fluorescence‐activated cell sorting (FACS). The level of peptide expression was quantified simultaneously, using a FLAG‐binding antibody conjugated to allophycocyanin (details in Methods).

To evaluate the TRAF6 MATH domain interaction motif space, we constructed a combinatorial library by introducing random residue variation around the core TIM6 element PxE. We used degenerate NNK codons to encode any of 20 amino acids at “x” positions in the sequence xxxPxExxx. The proline at position (+0) and the glutamate at position (+2) were held fixed to increase the proportion of binders in the library and to force a specific binding register to facilitate analysis and modeling. These sequences were presented in the context of flanking sequences from CD40; see Table S1 for details of the display constructs. To isolate cells displaying peptides that bound to the TRAF6 trimer, we carried out one round of initial enrichment using magnetic microbeads (see Methods). This procedure generated a smaller library, enriched in TRAF6 binders, that we designate MACSLib; this library was used as the input for subsequent enrichment experiments.

**FIGURE 2:**
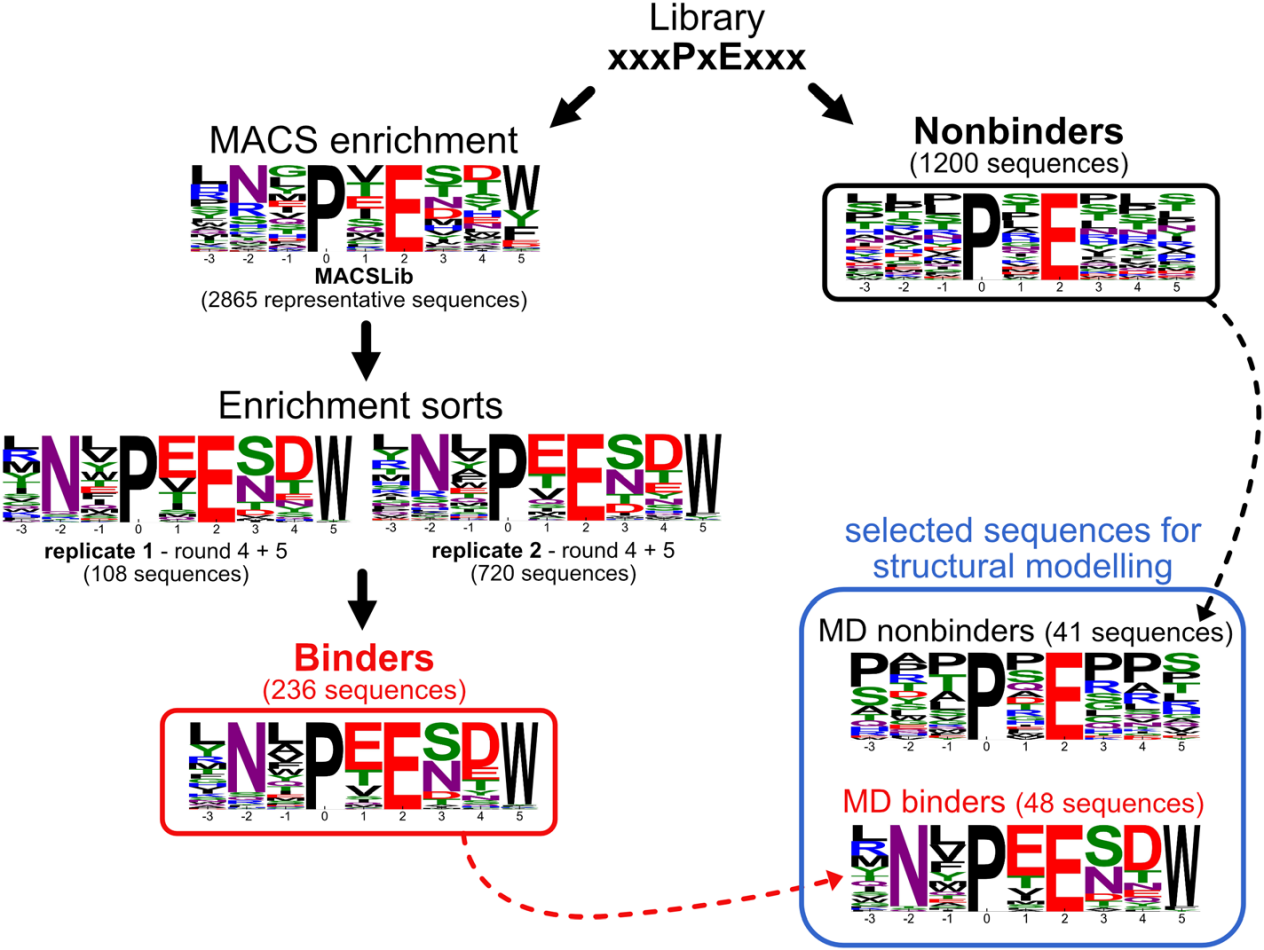
Sequence logos for TRAF6‐binding and nonbinding peptides. TRAF6 binders were identified by initial MACS enrichment followed by two replicate 5‐round FACS enrichment experiments. MACSLib and enrichment sort replicate logos are built from the unique sequences with read count ≥ 20 in the samples. A total of 2,865 sequences obtained after the MACS enrichment represent only a subset of the library at that stage but reflect the diverse residue content provided as input to the enrichment sorts. The final list of binders (red box) was generated by combining results from both replicates and further filtering for sequences that enriched across multiple rounds. Nonbinders were defined as sequences with read count ≥ 20 in the nonbinder pool (black box). Sequences of peptides selected for structural modeling are summarized in the blue box. Residue height in the logos represents the frequency of that residue in the sequence set. The number of sequences in each set is shown in parentheses.

To identify high‐affinity binders in MACSLib, two separate 5‐round enrichment sorts were performed using FACS to separate binding library members from nonbinding members (details in Methods). The stringency of the binding assay was gradually increased by using a lower concentration of T6cc for each round, from 300 to 3 nM. Following sorting, the population of binding cells in each round was deep sequenced to monitor the enrichment of individual sequences. The two replicate sorting experiments gave similar results, with sequences from rounds four and five reflecting similar preferred residues, indicating convergence of the selection process (Figure 2). In both replicate experiments, the three sequences LNLPEESDW, RNVPEESDW, and TNWPEENDW ranked among the top four binders based on sequencing read counts, and 14 of the top‐20 most‐represented sequences were the same in the two datasets. Examination of enriched sequences, particularly those in the final rounds, indicated a strong preference for Trp at position (+5). In addition, preferences were evident for Asn at (−2), Glu at (+1), Ser/Asn at (+3), and a polar or acidic residue at (+4). A final set of 236 high‐confidence TRAF6 binders was generated by taking the set of sequences present in rounds 4 and 5 of either replicate and filtering for sequences that enriched over at least 2 rounds of sorting (Figure 2 red box; see Methods for details). We also generated a population of nonbinders by collecting cells from the original unenriched library that gave a strong peptide‐expression signal but no TRAF6 binding signal. The logo for nonbinders did not show strong enrichment of any particular features (Figure 2, black box), and the diversity of the nonbinders confirmed that the input library included all 20 amino acids at each of the “x” positions.

To verify that the screening hits bound to TRAF6 in a concentration‐dependent manner, we performed single‐clone titration experiments. After titrating TRAF6 trimers into a clonal population of peptide‐displaying cells, we determined an apparent cell‐surface dissociation constant ($K_{d}^{*}$) by fitting the binding signal versus TRAF6 concentration to a standard binding model (Methods; Equation (1)). Binding between TRAF6 trimers and peptide‐displaying cells was multivalent, and the avidity enhanced the apparent dissociation constants. We measured $K_{d}^{*}$ for 14 peptides selected from the enrichment data as well as a peptide from CD40 with an affinity‐enhancing point mutation (KQEPQEIDF, here termed CD40*).^19^, ^28^ Interestingly, all of the top peptides from the enrichment bound TRAF6 with an apparent affinity tighter than the CD40* peptide, with some binding over 10‐fold tighter (Table 1 and Figure S1; see Methods). For example, RNVPEESDW, LNLPEESDW, and TNWPEENDW bound TRAF6 with $K_{d}^{*}$ values of 31, 46, and 84 nM, respectively, whereas CD40* bound with $K_{d}^{*}$ = 1.2 μM.

**TABLE 1:** Validation of binding for peptides enriched in the cell‐surface display screen

Peptide sequence	Single clone FACS *K*_d_* (μM)^a^	BLI *K*_d_ (μM)^b^
KQEPQEIDF (CD40*)	1.2 ± 0.21	240 ± 23
TNWPEENDW	0.084 ± 0.022	37 ± 6.9
LNLPEESDW	0.046 ± 0.0090	28 ± 3.1
RNVPEESDW	0.031 ± 0.0024^c^	24 ± 3.8

^a^ Single clone FACS *K*_d_* measurements were performed with trimeric TRAF6 (T6cc).

^b^ BLI *K*_d_ measurements were performed with monomeric TRAF6 (T6m).

^c^ Average of 2 replicates; in all other cases, the reported values are the average of 3 replicate binding curves ± the standard error of the mean.

**FIGURE 3:**
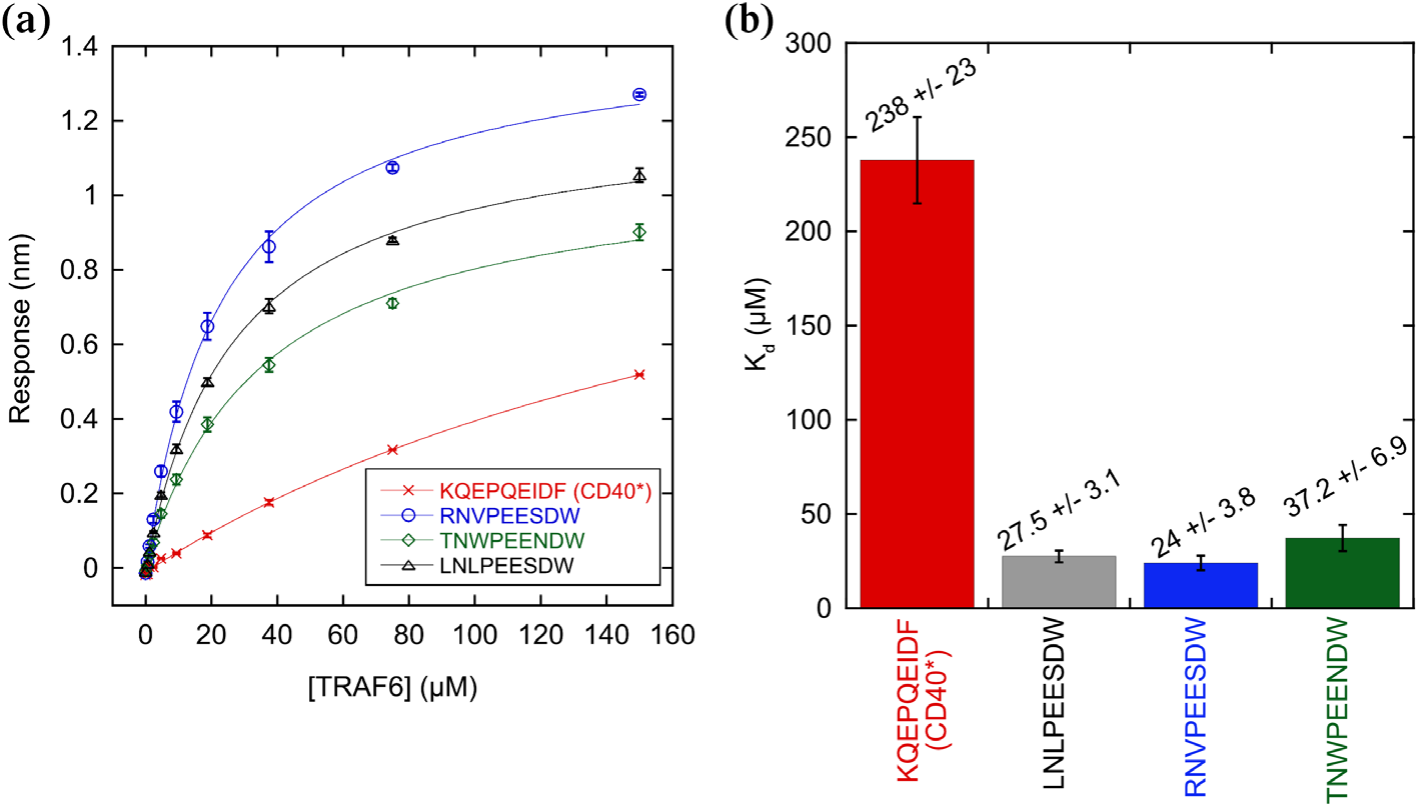
Biolayer interferometry (BLI) measurements of TRAF6 monomer in solution binding to different peptides on the BLI tip. (a) Binding signal is plotted against TRAF6 concentration and fit to a standard single‐site binding equation (Equation 1). Error bars are the standard error of the mean of three replicate measurements. (b) Average dissociation constant from three independently‐fit replicate binding curves. The error bars are the standard error of the mean. The dissociation constant fit for the CD40* peptide has a large error because the highest TRAF6 concentration used was 150 μM.

To further validate the cell‐surface interactions identified in the screen, we measured TRAF6‐peptide binding by biolayer interferometry (BLI), using purified monomeric TRAF6 MATH domain (construct termed T6m) in solution and purified peptides attached to the sensor tip (see Table S1 for construct details). By BLI, RNVPEESDW, LNLPEESDW and TNWPEENDW bound to TRAF6 with *K*_d_ values of 24.0, 27.5, and 37.2 μM, respectively, while CD40* bound with a *K*_d_ of 238 μM (Figure 3). The BLI data validate the cell‐surface display results and support the conclusion that top hits from the screen bind with higher affinity than CD40*, which is one of the tightest known TRAF6 peptide binders^19^ (Figure 3). Based on these observations, we conclude that despite the screening assay being performed in the environment of the cell surface, and in a multi‐valent context, enrichment sorting returned high‐affinity binders.

### 2.2 Structural modeling explains positive and negative binding determinants

For computational analysis of the structural determinants of TRAF6‐TIM6 binding, we chose a subset of high‐affinity binders identified from enrichment sorting (termed MD binders) and a subset of sequences designated as nonbinders (termed MD nonbinders) (see Methods and supplementary information). Figure 2 shows logos summarizing features of the two subsets of sequences.

**FIGURE 4:**
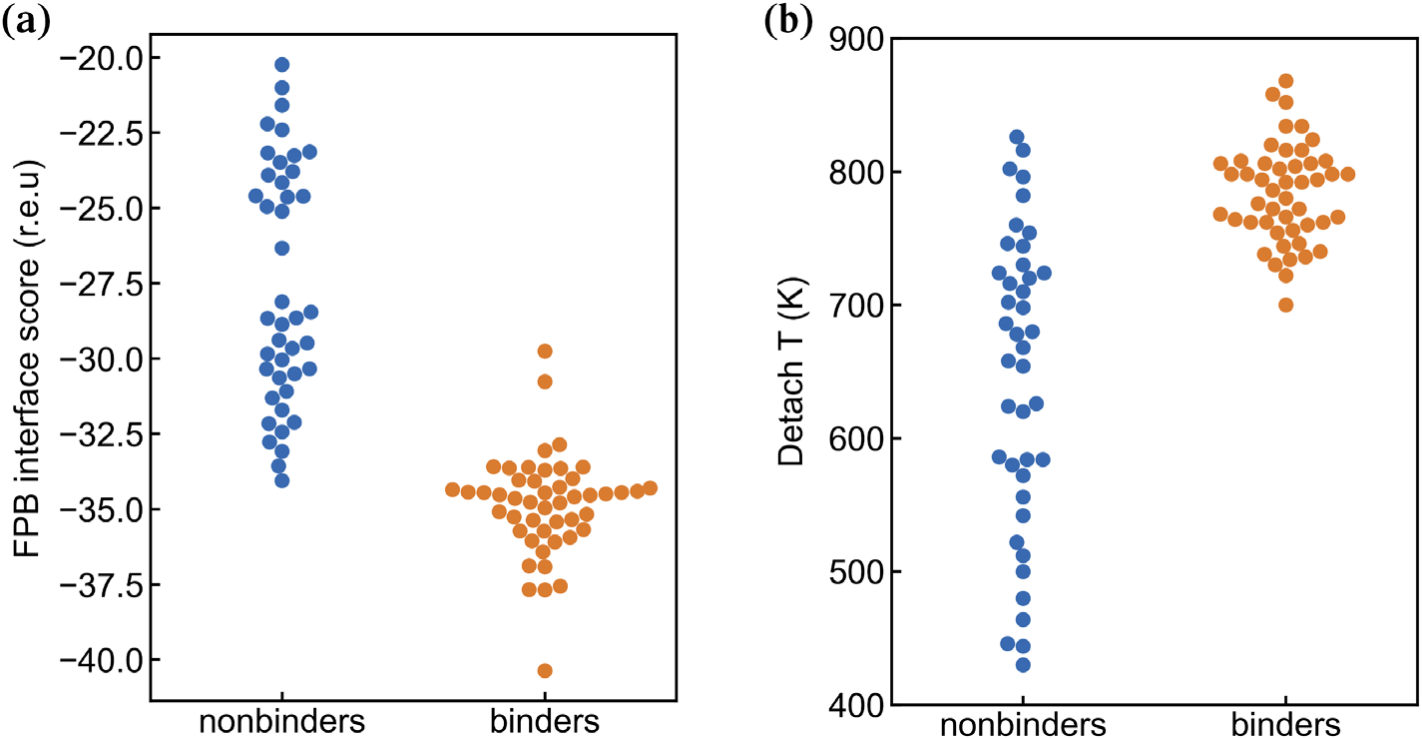
FlexPepBind (FPB) and Detach T scoring of TRAF6 MD binders and MD nonbinders. The FPB interface score (a) and Detach T (b) are plotted for a set of TRAF6 peptide binders (orange) and a set of nonbinders (blue) identified by high‐throughput screening.

We first tested whether FlexPepBind (FPB), a peptide modeling protocol in the Rosetta suite,^33^ could distinguish the MD binders from the MD nonbinders. As input to FPB, we prepared models of MATH domain‐peptide complexes using the structure of TRAF6 bound to CD40* (KQEPQEIDF) (PDB ID 1LB6) as a template.^19^ Starting from this initial docking position, the binding pose of the peptide was sampled, and the lowest interface score over all sampled poses was assigned to each peptide complex (see Methods for details). Figure 4a shows the score distributions for the 48 binders and 41 nonbinders by FPB score, which achieves a good separation of the two populations. CD40*, scored with the same protocol, gave an FPB interface score of −34.2, which is in the weaker end of the range of binding peptides, consistent with the affinity measurements discussed above.

Because 9‐residue peptides will sample an ensemble of conformations when bound to the TRAF6 domain, we also tested a molecular dynamics‐based protocol for evaluating peptide‐domain interactions. Starting with complexes modeled on the structure of TRAF6 bound to KQEPQEIDF, as described above, we computed a detachment temperature (Detach T) for each model, corresponding to the temperature at which the distance between the alpha‐carbon of TRAF6 Phe 471 and the center of mass of the peptide increased beyond 7 Å when the temperature was gradually increased from 300 K. Detach T, like FPB interface score, was able to separate binding peptides from most nonbinders, as shown in Figure 4b, with no binders giving Detach T values lower than 700 K.

**FIGURE 5:**
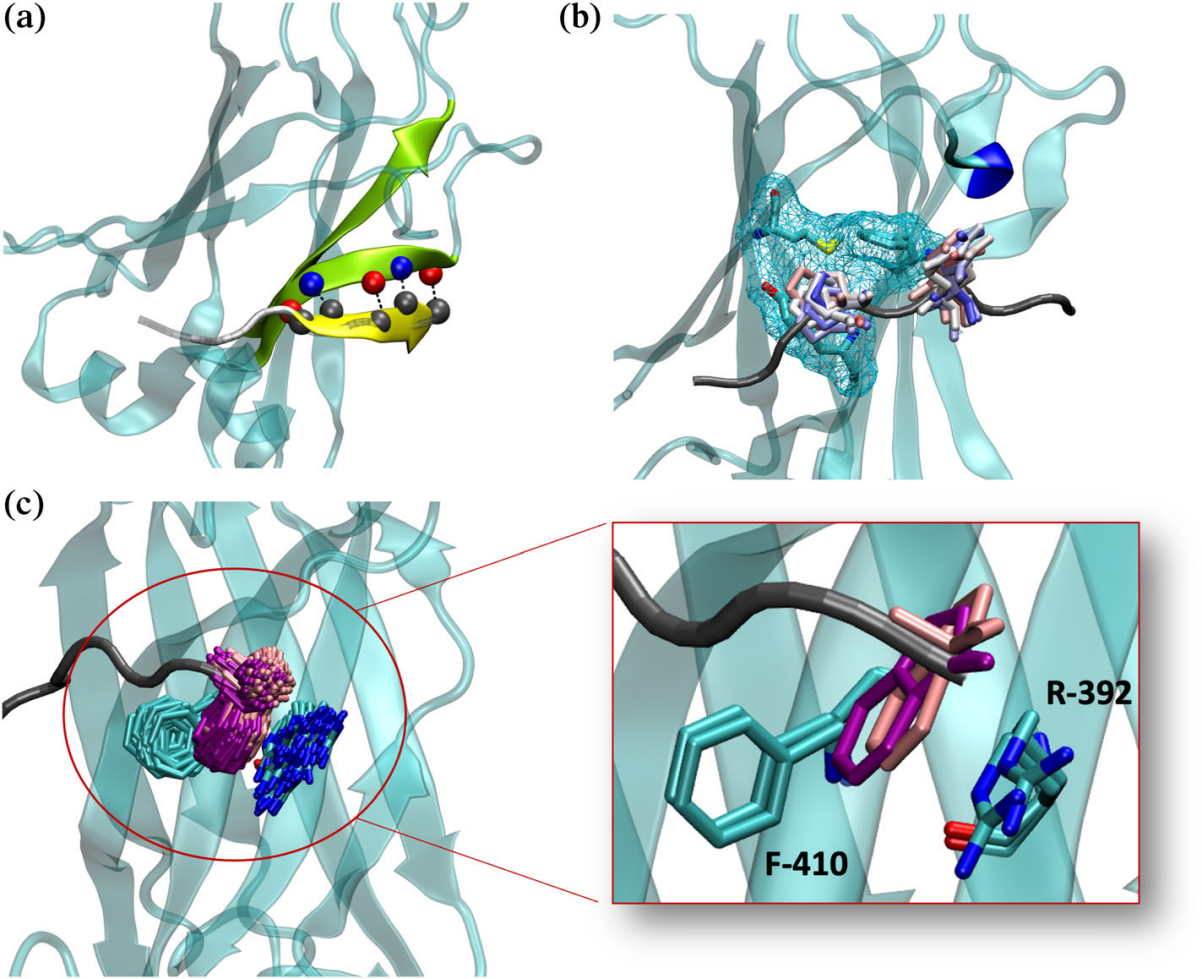
Structurally conserved features of high‐affinity complexes. (a) Five beta‐sheet hydrogen bonds involve main‐chain atoms of residues at positions (+1), (+3), (+5) (yellow), and residues 472, 470, and 468 in the MATH domain (green). The image shows a snapshot from a simulation of TRAF6 MATH in complex with the peptide LNLPEESDW. (b) Positions of Pro at (+0) and Glu at (+2) (sidechains in sticks) from different frames of the equilibrated MD simulation of the peptide LNLPEESDW bound to TRAF6 MATH (color scale: red‐white‐blue for snapshots from the beginning‐middle‐end of the equilibrated part of the simulation). Pro binds into the pocket shown with cyan mesh, and Glu caps the short helix marked in blue. (c) The two most populated clusters for Trp conformations at position (+5) for 65% of the high‐affinity binders. This sidechain arrangement allows simultaneous pi–pi interaction with Phe 410 and cation–pi interaction with Arg 392. The expanded region highlights snapshots from the two most common conformations.

To explore the structural origins of sequence trends apparent in our enrichment sorting results, we used molecular dynamics simulations to analyze TRAF6 complexes with peptide ligands CD40*, each of the 48 MD binders, and each of the 41 MD nonbinders (see Methods). With CD40* and all MD binders, simulations showed the persistence of 5 hydrogen bonds that positioned the peptide as an extension of the beta‐sheet in the MATH domain, as seen for CD40* in PDB structure 1LB6 (Figures 1c and 5a). The hydrogen bonds involved backbone atoms of residues in positions (+1), (+3), and (+5) that were highly stable during all 80 ns of equilibrated‐MD simulation. Invariant TIM6 residues Pro at (+0) and Glu at (+2) also preserved their crystallographic positions throughout all trajectories, with only minor displacements (Figure 5b). Pro at (+0) was accommodated in the pocket created by residues Phe 471, Met 450, and Tyr 473, while the negatively charged Glu at (+2) capped a 3–10 helix formed by residues Leu 456, Leu 457, and Ala 458 (Figure 5b) in the MATH domain.

Trp at (+5) was present in most of the binders obtained from enrichment sorting, even though this residue is not common in known native interaction partners of TRAF6 (Figure 1a). Indeed, only 14 out of 236 binder sequences identified in the enrichment screen did not have tryptophan at position (+5). Our simulations showed different conformations for the Trp sidechain. Most frequently, the indole group was inserted into the receptor pocket (Figure 5c), allowing for simultaneous pi‐pi interaction with Phe 410 and cation‐pi interaction with Arg 392. This conformation represented the most populated cluster for 65% of the MD peptides and resembles the conformation of Phe at position (+5) in the complex of CD40* bound to TRAF6 MATH (Figure 1d).^19^ In particular, clustering the (+5) Trp conformations from simulation frames by RMSD showed that more than 60% of the conformations were within 1 Å of the sidechain arrangement shown in Figure 5c. We also observed structures in which the Trp indole was flipped out of the pocket but maintained a binding interface, including backbone H‐bond interactions with Pro 468 and occasional pi–pi or cation–pi interactions with Phe 410 or Arg 392. Such conformations were shared among 20% of the MD binders. The remaining 15% of the MD binders showed unclustered Trp (+5) conformations in which the backbone was still involved in an H‐bond interaction with Pro 468, but the indole group was flipped out and did not contact the MATH domain.

**FIGURE 6:**
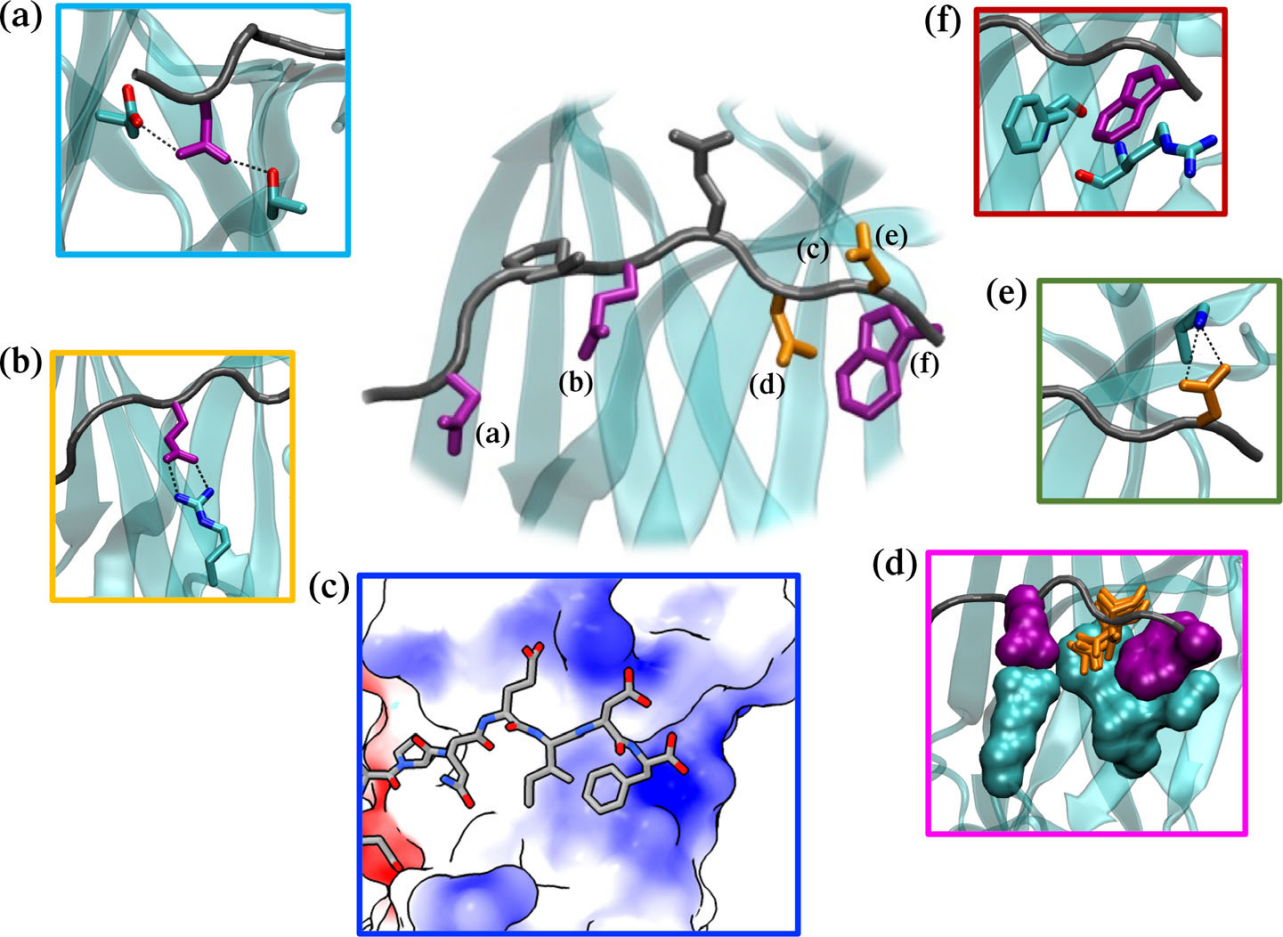
Overview of the most significant contacts between the highest affinity binders and the TRAF6 MATH domain, as captured in molecular dynamics simulations. Panels (a)–(f) illustrate specific interactions discussed in the text. Key peptide residues are represented in sticks: grey for Pro at (+0) and Glu at (+2), purple for residues at positions that favor a particular amino acid, and orange for residues at positions that favor a group of amino acids with similar features. All of the highlighted interactions are present for >30% of simulation time for all MD binders. (a) Asn at (−2) can simultaneously form H‐bonds with Glu 448 and Thr 475. (b) Glu at (+1) forms a bi‐dentate salt‐bridge interaction with Arg 402 when both sidechains are fully extended. (c) Residues at positions (+3), (+4), and (+5), are located in an electrostatically positive region (as indicated by blue coloring), PDB ID 1LB6. (d) Interactions involving residues at (+1) and (+5), shown as space‐filling Glu and Trp in purple, along with MATH domain residues 402, 410, 392, and 394 (space‐filling, cyan), narrow the pocket at position (+3) so that small residues, such as the Asn pictured in orange sticks, are preferred at this site. (e) Residues at position (+4) face solvent, and acidic residues at this site, such as the pictured Asp, can form a salt bridge with Lys 469. (F) Trp at (+5) engages in edge‐to‐face pi‐pi and cation‐pi interactions with residues Phe 410 and Arg 392, respectively.

The preference for Asn at (−2) in binders from the screen can be explained by its sidechain interactions with nearby TRAF6 residues. In all of the MD binders, the Asn formed a stable interaction with the backbone of Thr 475 on TRAF6 for >40% of simulation time. In more than 70% of the MD binders, the Asn also formed a hydrogen bond with Glu 448 on TRAF6 for >30% of the simulation time (Figure 6a). Longer residues at (−2) (e.g., Gln) were unable to interact with both TRAF6 amino acids. This interaction pattern appears to be important for high‐affinity binding: Asn at (−2) is present in 190 of the 236 binders from the enrichment.

A preference for Glu at position (+1) can be explained by a salt–bridge interaction that this residue forms with Arg 402 in the MATH domain (Figure 6b). Despite a Cα‐Cα distance of ~10 Å, the Arg 402 sidechain can form a salt bridge with the (+1) Glu when both are fully extended toward one another. In the simulations, this interaction was stable for more than 80% of equilibrated trajectory time and was completely missing for peptides in which Glu is substituted with Asp due to the shorter sidechain of the smaller residue.

At position (+3), interactions involving residues at (+1) and (+5), and the positions of MATH domain residues 392, 394, 402, 410, and 474, narrow the pocket, providing an explanation for why small residues, such as Asn and Ser, are preferred at this site (Figure 6d). Residues at position (+4) are less sterically constrained and some can form a hydrogen bond or salt bridge with surface Lys 469 (Figure 6e); all MD binder peptides with Glu at (+4) formed a salt bridge with Lys 469 in more than 60% of simulation frames.

Analyzing our models of the MD binders helped explain why many nonbinders did not form tight interactions with TRAF6, despite including the conserved Pro at (+0) and Glu at (+2). At positions (+1), (+3), and (+5), the MD binders make hydrogen bonds that complete a beta‐sheet with TRAF6. Proline residues are disfavored in beta structures because they lack the required NH group for this interaction and prefer backbone dihedral angles far from the typical range in β‐sheets.^34^ Thus, Pro at any position between (+1) and (+5) is expected to be highly unfavorable. Indeed, Pullen et al. showed that mutation to proline at any of these positions in a peptide from CD40 (sequence KQE**P**Q**E**INFPDDLP) abrogated binding in peptide array experiments.^28^ A total of 386 of the 1,200 nonbinders (32%) identified in our screen have such a substitution, which is likely sufficient to prevent high‐affinity binding. None of the 236 binders contain a proline at these positions. Furthermore, the TRAF6 MATH domain is electrostatically positive near positions (+3), (+4), and (+5) (Figure 6c), suggesting that positively charged residues would be destabilizing at these sites. Indeed, Arg or Lys are found at one or more of these positions in 287 of 1,200 nonbinders (24%) but in only 2 of 236 binders (0.8%). Mutations by Pullen et al. confirm that these substitutions disrupt binding at these three positions in the context of a CD40 peptide.^28^ Steric constraints at position (+3) are further expected to disfavor medium or large residues at this site. Consistent with this, residues Q, H, I, L, F, Y, or W are found at position (+3) in 396 of the 1,200 nonbinders (33%) but in only 4 of the 236 binders (2%). Overall, 851 of the 1,200 nonbinders (71%) have at least 1 of the unfavorable sequence features described above (see Table 2 for summary). The nonbinders also lack key residues that form stabilizing interactions in the highest affinity binders. Only 158 of the 1,200 nonbinders (13%) contain Asn at (−2), Glu at (+1), Asp at (+4), or Trp at (+5), while all of the 236 binders contain at least one of these interactions. Only 7 of 1,200 nonbinders (0.6%) contain two or more of these stabilizing residues, while 224 of 236 binders (95%) contain two or more of these interactions.

**TABLE 2:** Fraction of binder and nonbinder sequence sets with certain sequence features

Sequence feature	Binders	Nonbinders
Proline any position between (+1) and (+5)	0/236	386/1200
Positive charge (R, K) at (+3), (+4), or (+5)	2/236	287/1200
Large/medium residues (Q, H, I, L, F, Y, W) at (+3)	4/236	396/1200

### 2.3 Candidate TRAF6 interaction motifs in the proteome do not share the sequence features of the top screening hits

**FIGURE 7:**
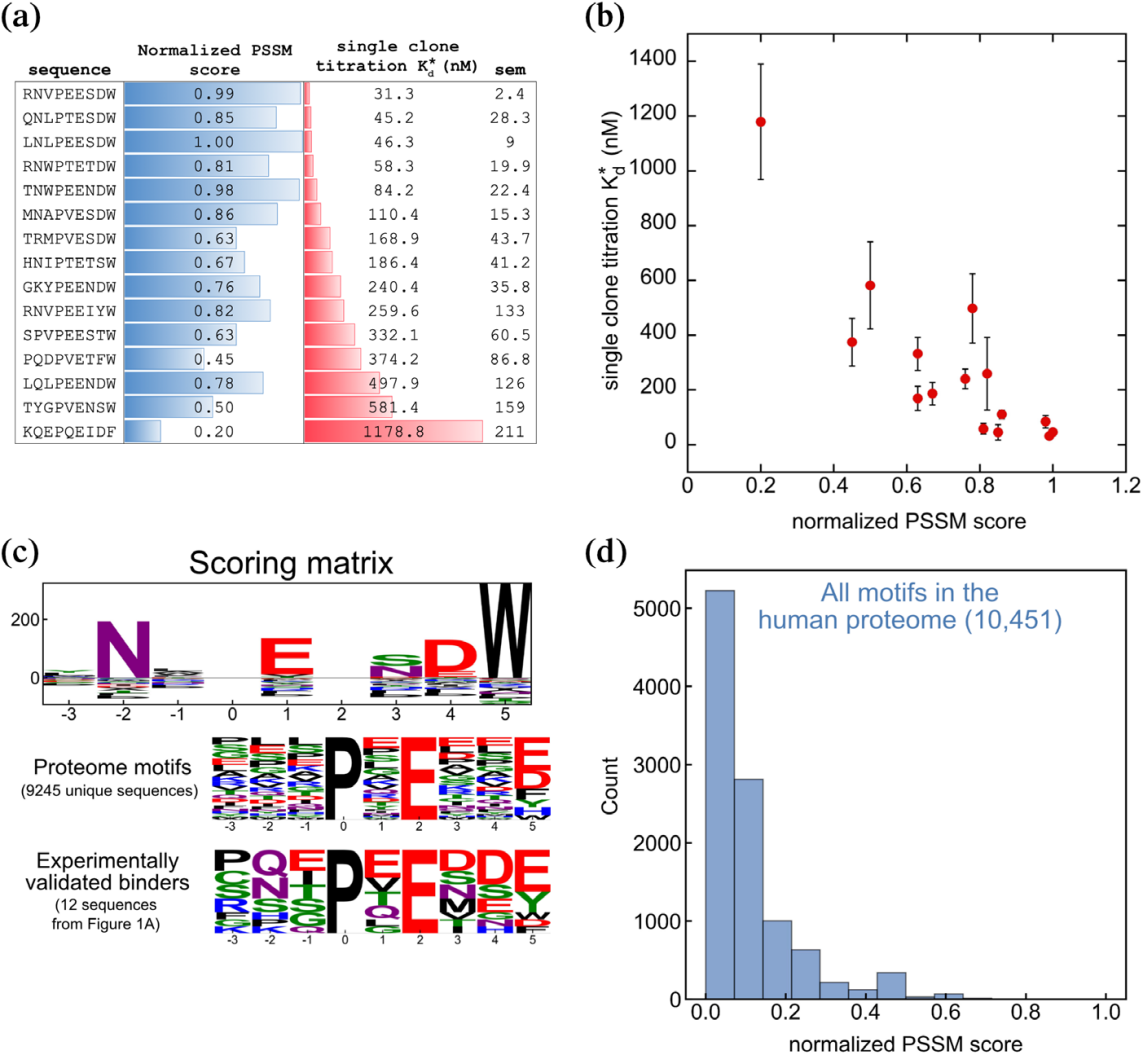
TRAF6 motif scoring. (a and b) The PSSM scores of selected TRAF6 binding peptides are correlated with their apparent cell‐surface binding affinity for TRAF6. (a) Reported dissociation constants are the average of fits to 2–3 replicate titrations. The standard error of the mean (*SEM*) is reported for each sequence. (b) Correlation between $K_{d}^{*}$ and PSSM score (data from a). Error bars are the standard error of the mean of 2–3 replicates. (c, top) Position‐specific scoring matrix generated from the screening data and used to score candidate binding motifs. (c, bottom) Sequence logos of all unique TRAF6 motif matches (motif: xxxPxExx[FYWHDE]) in the human proteome compared to experimentally validated TRAF6 MATH domain binders. (d) Distribution of normalized PSSM scores of all TRAF6 motif matches in the proteome, using the matrix shown in the top of panel c.

We investigated whether any human proteins contain close matches to the high‐affinity sequences identified by screening. We defined a position‐specific scoring matrix (PSSM) to score candidate TRAF6 interaction motifs based on how well they match our top binders. We used pLogo,^35^ a log‐odds‐based method, to construct the PSSM, using the 236 binding sequences from the enrichment as the foreground and the 1,200 nonbinder sequences as the background. The nonbinder sequences were considered a fair approximation of the sequence composition of the input library, assuming that TRAF6 binders are rare in the library. Indeed, we do not observe any apparent residue preferences in the nonbinder set (Figure 2). To test if the PSSM score of a sequence represents how well that peptide binds to TRAF6 on the cell surface, we scored the sequences used in single clone titrations. Scores were normalized to the range 0 to 1, with 0 the lowest and 1 the highest possible PSSM score. We found that PSSM score is correlated with apparent cell‐surface affinity, suggesting that our model is a good predictor of TRAF6 binding within this sequence space (Figures 7a,b).

The PSSM was used to score TRAF6 motif matches in the human proteome to identify SLiMs with the potential to bind with high affinity. TIM6 matches (10,451 hits total) were obtained using the SLiMSearch tool^36^ (regular expression: …P.E…[FYWHDE]). The logo of hits is shown in Figure 7c, along with a logo of the experimentally validated TRAF6 binding peptides from Figure 1a. Figure 7d shows the distribution of PSSM scores for the sequences retrieved using SLiMSearch.^36^ Notably, no sequences in the proteome occupy the sequence space favored in the screen (i.e., no sequences have a high score). The highest‐scoring sequence in the proteome is FNEPEENFW, with a score of 0.85. Only 4 motifs in the proteome have a PSSM score above 0.75, and only 1 of those is predicted to be disordered by IUPred (IUPred >0.4^37^). Additionally, the motifs in the proteome that are experimentally validated to bind to the TRAF6 MATH domain have low PSSM scores (Table S3).

Despite the low scores for proteome sequences, we explored whether the screen‐based PSSM could be used in conjunction with other metrics to identify proteome sequences with the potential to interact with TRAF6 with high affinity. We constructed a table from the proteome motif matches that includes a variety of scores and filters for each hit, compiled from multiple sources (Table S4; see the supplementary information for details). We included indicators of whether the motif is predicted to be structurally accessible for binding (IUPred score^37^ and AlphaFold pLDDT score^38^, ^39^, ^40^), whether the candidate protein is involved in similar biological processes as TRAF6 (based on shared Gene Ontology (GO) annotations with TRAF6, from SLiMSearch),^36^, ^41^, ^42^ whether the protein has been annotated to interact with TRAF6 (HIPPIE database^43^), and whether the motif has any unfavorable sequence features identified in our structural analysis (Table 2). Applying filters based on these criteria narrowed the list of potential biologically relevant TRAF6 motifs in the proteome from ~10,000 to ~1,000 sequences matching the xxxPxExx[FYWHDE] motif. Among these candidate motifs, we chose a few with high PSSM scores, indicating potentially high affinity, for further analysis (Table S4).

One of the highest‐scoring hits in the proteome is the sequence QNFPVESDW (PSSM score = 0.85) from RNF103. RNF103 acts as an E3 ubiquitin‐protein ligase that is localized to the ER membrane; it is involved in the ER‐associated degradation (ERAD) pathway.^44^ This sequence has the highly favorable residues Asn and Trp at positions (−2) and (+5), respectively. The sequence also contains favorable Ser and Asp residues at (+3) and (+4), respectively. The TRAF6 motif match is not predicted to be disordered by IUPRED. However, the average AlphaFold pLDDT score of the motif is 38.7 (corresponding to predicted disorder) and the motif residues appear accessible in the AlphaFold‐predicted structure.^38^, ^39^, ^40^ Although RNF103 localizes to the ER membrane, the candidate motif (positions 474–482) maps to the cytosol, given its location between the last transmembrane helix and the cytosolic RING domain of RNF103.

The sequence GMGPVEESW starts at position 350 in RIPK1. The sequence has a PSSM score of only 0.46, but has a highly favorable Trp at (+5) and lacks any of the major unfavorable sequence features identified by structural modeling (Table 2). RIPK1 is a serine–threonine kinase involved in regulating TNF‐mediated apoptosis, necroptosis, and inflammatory pathways.^45^ It has been annotated as a TRAF6 interaction partner in the HIPPIE database, but the details of the interaction are unknown. RIPK1 has been found to bind to other TRAF proteins^46^ and also to TICAM1.^47^ We speculate that RIPK1 may interact with TRAF6 via the MATH domain engaging this short segment.

Another hit with potential for biological significance is the sequence GNFPEENND, which spans positions 1,065–1,073 in the leptin receptor. This sequence contains an Asn at (−2), Glu at (+1), and Asn at (+3), which are all favorable residues according to our model. It has a PSSM score of 0.49. The leptin receptor binds leptin, which is secreted from adipose cells. In obese mammals, leptin levels are elevated, leading to chronic low‐grade inflammation.^48^ TRAF6 is a well‐known regulator of the inflammation response, suggesting a possible link between the two pathways.

SLiMs are known to evolve rapidly,^49^ so although conservation of a motif can support its functional relevance, lack of conservation does not necessarily indicate that a motif is not functional. It is notable that the motif instances in RNF103 and the leptin receptor are not highly conserved across species (Figure S3). For RIPK1, the tryptophan at (+5) in the motif is not conserved beyond mammals. However, the presence of a TRAF6 motif in this region of RIPK1 appears widely conserved across species; the TRAF6 motifs in other species typically include acidic glutamate at (+5). Interestingly, the position of the motif within RIPK1 also varies among species (Figure S3), which has been previously observed for SLiM evolution.^49^ Experimental follow‐up will be required to assess whether any of the proteome hits interact with TRAF6 in a biological context. Overall, our conclusion is that the proteome contains few sequences that match our screen‐derived PSSM, and most native sequences with high scores do not appear to be good interaction candidates based on other metrics. Biology does not use the sequence space of highest affinity for native TRAF6 interactions.

## 3 DISCUSSION

The discovery of TRAF6 interaction partners over decades of experimental research has led to a definition of the TRAF6 MATH domain binding motif as xxxPxExx[FYWHDE]. This was arrived at by compiling aligned, verified TRAF6 binding sequences and identifying their common sequence features.^19^, ^23^, ^24^, ^25^, ^26^, ^27^, ^28^ In this work, we explored the TRAF6 motif sequence space systematically, using cell‐surface screening of a combinatorial library that presented the core PxE motif flanked by random residues. The top hits obtained from this screen bound with affinities comparable to or higher than known TRAF6 interaction partners reported in the literature.^19^, ^24^ Analysis of screening hits highlighted which residues were most preferred at each position and identified features that differentiate binding sequences from nonbinding sequences among protein segments that contain the core element PxE.

Two different methods of structure‐based modeling could distinguish the best‐binding peptides from the background, and we used molecular dynamics simulations to study the bound ensembles of diverse binders. This analysis provided a structural explanation for the residue preferences observed in our screening data. In particular, in all high‐confidence binders that we analyzed, Asn at position (−2) can form favorable interactions with Glu 448 on TRAF6, Glu at position (+1) can form a salt bridge with Arg 402 on TRAF6, and Trp at position (+5) can form pi‐pi interactions with Phe 410 and cation‐pi interactions with Arg 392. Our structural analysis also highlighted negative design elements that can disfavor PxE‐containing segments binding to TRAF6 MATH. The logo of nonbinders in Figure 2 does not indicate any strong features, but our data support a model in which a variety of sequence features, including proline in positions (+1) to (+5), a large residue at position (+3), or a positively charged residue at (+3), (+4), or (+5) can disfavor binding. Thus, within this 9‐residue stretch that includes PxE, several positive features and the absence of a variety of negative‐design elements are important for making a functional TRAF6 binder.

The sequence preferences observed in this study, determined using a large and diverse library, can be compared with point mutations in the native CD40 TIM6 peptide that were analyzed using peptide SPOT arrays.^28^ Overall, there is good agreement between the two studies regarding the favorable and unfavorable residues for binding. Contrary to our observations, however, the SPOT array suggests that large residues (H/I/L/F/Y/W) are tolerated at (+3), in the context of the CD40 peptide.^28^ This discrepancy could arise from epistasis between positions in the motif. For example, Trp at (+5) (as opposed to Phe, in CD40) may place stricter spatial constraints on the pocket at position (+3), such that large residues are occluded from position (+3) when there is a Trp at (+5), but not when the (+5) position is Phe (as in CD40). In support of this hypothesis, the CD40 SPOT array shows reduced binding when W is substituted for F at the (+5) position. Foight et al. also found coupling between mutations, and a sensitivity of mutations to motif context, in the interactions of peptides with TRAF2, TRAF3, and TRAF5 MATH domains.^3^

Our analysis of the proteome revealed that no sequences map to the high‐affinity, TRAF6 binding sequence space that we identified using cell‐surface display, and that our resulting PSSM is not a good predictor of endogenous TRAF6 binding sequences. Indeed, most of the well‐studied peptides from verified TRAF6 MATH domain binders lack the features of the highest‐affinity binders identified in the screen or only contain 1 or 2 of the most favorable interactions we identified (Figure 1a, Table S3). Only 3/12 have an Asn at (−2), and only TICAM1 contains Trp at (+5). Our findings imply that the core binding motif is either not under selection for high affinity, or high affinity is detrimental to TRAF6 function.

Other library‐based studies have found enrichment of hydrophobic residues in binders of protein domains that does not reflect the composition of the native binding partners.^2^, ^50^ The distinct properties of native vs. library‐selected binders could be due to natural selection for binding specificity, solubility, or peptide intrinsic disorder, rather than affinity. Weak SLiM binding that allows for transient and short‐lived interactions can also provide advantages.^51^ For example, complexes that use multiple weak interactions rather than one higher‐affinity binding site provide opportunities for regulation, and enhanced specificity, as is the case for tandem recognition of SLiMs by SH2, SH3, WW, and other domains.^52^, ^53^, ^54^, ^55^ The TRAF proteins provide a different example of the benefits of weak binding for signaling. TRAF6 uses avidity to significantly enhance binding affinity to oligomeric receptor proteins. In concentration regimes where the binding affinity of a single‐motif peptide is not significant, receptor oligomerization can trigger TRAF6 trimers binding to three or more motifs in the tails of clustered cytoplasmic receptors, which then promotes ubiquitylation that propagates the signal further downstream.^9^ Artificial oligomerization of TRAF6 alone is sufficient to activate signaling through certain pathways, implying that TRAF6 oligomerization is a key part of the signal transduction.^29^ In this scheme, conserving a weak, fast‐exchanging interaction between individual motifs and monomeric MATH domains is likely important for supporting rapid, ligand binding‐dependent assembly and disassembly of a TRAF6 signaling complex.

When profiling the binding preferences of a SLiM‐binding domain using high‐throughput screening experiments, different libraries may have different applications. For identification and prediction of new interaction partners in the proteome, biologically relevant libraries, such as a library of tiled sequences from the proteome, are likely more effective than a randomized library.^4^, ^30^ In contrast, for the purpose of synthetic biology or inhibitor design, a randomized library has the potential to better identify high‐affinity sequences that are more likely to out compete native binders.

High‐affinity TRAF6 binders isolated in this work can serve as lead peptides for inhibitor development. TRAF6 signaling is implicated in inflammation and cardiovascular disease.^12^, ^13^, ^18^ Targeting TRAF6 MATH is reported to improve insulin sensitivity in obese mice, improve heart function in mouse models of non‐ischemic cardiac failure, reduce atherosclerosis, and inhibit osteoclastogenesis and bone resorption.^14^, ^15^, ^16^, ^17^ A RANK peptide attached to a protein transduction sequence to promote cell entry is currently sold commercially as a TRAF6 inhibitor (e.g., Novus Biologicals NBP2‐26506).^16^ The reported affinity of a RANK peptide with sequence RKIPTEDEY for TRAF6 is 78 μM, determined by isothermal titration calorimetry.^19^ The same study reported a *K*_d_ of 84 μM for the CD40* peptide, and we showed that peptides from our screen bind ~10‐fold tighter than CD40* (Figure 3). Thus, peptides from our screen, possibly further optimized by adding an optimal flanking sequence, can serve as higher‐than‐native‐affinity inhibitors. Having a broad range of peptide sequences that can disrupt TRAF6 binding, as we have generated here, can support further efforts to develop inhibitors with desirable properties, such as low immunogenicity and cell permeability.

## 4 MATERIALS AND METHODS

*Vectors*, *bacterial cells*, *and cloning*: The expression constructs and cell surface display constructs are detailed in Table S1. The TRAF6 trimeric construct, here termed T6cc, consisted of residues 310–504 of human TRAF6 (including the MATH domain and coiled‐coil trimerization domain), an N‐terminal BAP tag for biotinylation, and a hexahistidine tag for purification. The construct was expressed using a pDW363 vector to ensure biotinylation. A monomeric TRAF6 construct lacking the trimerization domain and BAP tag, here termed T6m, consisted of residues 350–501 of human TRAF6 and a hexahistidine tag. The construct was expressed in a pDW363 vector, although the lack of a BAP tag ensured no biotinylation of this protein. SUMO‐peptide fusion constructs contained a BAP tag, hexahistidine tag, and SUMO tag. The construct was expressed in a pDW363 vector to ensure biotinylation. *E. coli* strains BL21(DE3), DH5α, and MC1061 were used for protein expression, cloning, and surface display, respectively. For bacterial surface display of TRAF6‐binding peptides, the eCPX vector designed by the Daugherty group^56^ was modified at the C‐terminus to append a FLAG sequence, a linker containing a SfiI site, and the CD40* peptide sequence, which included 9 residues resolved in the X‐ray structure of CD40* bound to TRAF6 (PDB ID: 1LB6^19^) plus 8 flanking residues on each side of this core region. The CD40*‐derived sequence used was PTNKAPHPKQEPQEIDFPDDLPGSNT.

*Mutant library construction*: The library was constructed using primers (from IDT) with NNK codons included in positions marked “x” the motif xxxPxExxx, such that the theoretical size of the library was 20^7^ = 1.28 * 10^9^ unique members. The variable sequence was flanked by SfiI restriction sites for cloning. In parallel, a linear vector for cell‐surface display containing the constant sequence of the display construct, with SfiI sites matching the library insert, was amplified by PCR. The insert and linear vector fragments were purified by PCR Cleanup Kits (Genesee Scientific) before SfiI digestion. Following purification of the digested fragments, a 5:1 ratio of insert: vector was added to a 200 μl T4 DNA Ligase (New England Biolabs) reaction and then incubated for 16 hours at 4°C. The ligated mixture was electroporated into fresh electrocompetent MC1061 cells in four separate transformations. Transformed cells were transferred into 10 ml warm Super Optimal Broth media with 20 mM glucose (SOC media) and incubated at 37°C for 1 hour. The 10 ml culture was then added to 1 L of LB + 25 μg/ml chloramphenicol and grown to an OD600 of 0.6–0.8 before centrifugation and resuspension in LB + 20% glycerol for freezing for storage.

*Protein purification and preparation*: T6cc was co‐expressed with the biotin ligase BirA (from the pDW363 vector) in BL21(DE3) *E*. *coli* for 5 hours at 37°C. Media was supplemented with 0.05 mM D‐(+)‐biotin. The protein was then purified using Ni^2+^‐NTA affinity chromatography followed by gel‐filtration chromatography into a final buffer of 20 mM Tris pH 8.0, 150 mM NaCl, 5% glycerol, 1 mM DTT. Purified protein was concentrated before storing at −80°C in aliquots for later use. Concentrations of T6cc are reported as monomer concentrations. For solution binding studies, a monomeric variant of TRAF6 (T6m) was expressed in Rosetta2(DE3) cells overnight at 18°C and purified similarly to T6cc. T6m was purified into a final buffer of 50 mM Tris pH 8.0, 180 mM NaCl, 5% glycerol, and 1 mM DTT. SUMO‐peptide fusion proteins were co‐expressed with the biotin ligase BirA (from the pDW363 vector) in Rosetta2(DE3) *E. coli* for 5 hours at 37°C. Media was supplemented with 0.05 mM D‐(+)‐biotin. The protein was purified by Ni^2+^‐NTA affinity chromatography followed by gel‐filtration into a final buffer of 20 mM Tris pH 8.0, 150 mM NaCl, 1 mM DTT, and 10% glycerol.

*Magnetic bead presorting (MACS)*: To generate TRAF6‐bound beads, 2 ml of vortex‐mixed Invitrogen DynaBeads™ Biotin Binder beads were incubated with T6cc (33 pmol biotinylated T6cc/10 μl beads) for 2 hours at 4°C and then washed in PBS buffer, as described by Angelini et al.^57^ The TRAF6‐decorated beads were then added to cultures of induced cells expressing the peptide library (induced with 0.2% w/v arabinose for 2 hours at 37°C). After incubation for 3 hours at 4°C, beads were magnetically isolated for 60 seconds before aspiration and replacement of PBS buffer. Beads were then gently shaken in the fresh buffer for 5 minutes at 4°C. The bead wash cycle was repeated 7 times before beads were placed in LB media for growth overnight. 100 μl of the final growth stock was serially diluted on LB + agar +25 μg/ml chloramphenicol plates. Colony‐forming units were tabulated to back‐calculate the number of cells in the MACS‐sorted library, which yielded 1.42 * 10^5^ cells.

*Bacterial FACS preparation*: For enrichment sorts and single‐clone FACS cell surface titrations, 5 mL cell cultures were grown overnight at 37°C in LB + 25 μg/mL chloramphenicol and 0.2% w/v glucose. The next day the culture cell density was measured by OD_600_, and approximately 3.25 * 10^5^ cells of each stock were isolated for new growth in 5 mL LB. Upon reaching an OD_600_ of 0.5–0.6, cells were induced with 0.2% w/v arabinose for 2 hours at 37°C. Density was again measured, and cells were pelleted by centrifugation and resuspended in PBS + 0.5% BSA. Cells were then aliquoted into a 96‐well Multi‐Screen HTS® GV sterile filtration plate (2 x 10^7^ cells per sample) and washed with fresh PBS + 0.5% BSA. Cells were then incubated in 30 μl of αFLAG‐APC [PerkinElmer] (prepared at a 100:1 dilution in PBS + 0.5% BSA) at 4°C for 15 min. Next, cells were resuspended in 50 μl of TRAF6 solution (25 μl PBS + 0.5% BSA mixed with 25 μl of the chosen TRAF6 concentration) and incubated at 4°C for 60 min. Following a wash with 200 μl PBS + 0.1% BSA, cells were resuspended in 30 μl streptavidin‐PE (SA‐PE) [ThermoFisher] (prepared at a 100:1 dilution in PBS + 0.1% BSA) and incubated at 4°C for 15 minutes. Cells were then washed in 200 μl PBS + 0.1% BSA, resuspended in another 200 μl PBS + 0.1% BSA, and placed on ice prior to FACS analysis or sorting. FACS analysis was performed using an HTS Canto II instrument and sorting took place on a FACS Aria III cell sorter (BD Biosciences). Sorted cells were collected in 1.5 ml microcentrifuge tubes containing 500 μl Luria‐Bertani media with 25 μg/ml chloramphenicol.

*Single‐clone titration experiments*. For single‐clone titration experiments, samples for FACS analysis were prepared as described above using eight concentrations of TRAF6 for each clone: 0 nM, 3 nM, 10 nM, 30 nM, 100 nM, 300 nM, 1 μM, 3 μM. Binding curves were generated by plotting the mean PE value vs. TRAF6 concentration and fit to the following equation to determine a $K_{d}^{*}$ value:

(1) $$y=F_{\text{init}}+\left(F_{\mathrm{sat}}-F_{\text{init}}\right)\left(\frac{x}{x+K_{d}^{*}}\right)$$

where *y* is the mean PE fluorescence value and *x* is the concentration of TRAF6. *F*_init_, *F*_sat_, and *K*_d_ were treated as floating parameters; *F*_init_ is the *y* value in the absence of TRAF6 and *F*_sat_ is the *y* value at which the binding curve saturates. Although the cell‐surface binding data fit well to a hyperbolic binding equation, this assay is not likely to be at equilibrium, and we discourage interpretation of the apparent binding constant $K_{d}^{*}$ as a true equilibrium dissociation constant.

*Biolayer Interferometry (BLI)*: BLI experiments were carried out on an ﻿Octet Red96 instrument (ForteBio). Streptavidin‐coated tips (ForteBio) were pre‐incubated for 10 min in BLI buffer (20 mM Tris pH 8.0, 207 mM NaCl, 1 mM DTT, 1% Glycerol, 0.1% BSA, and 0.1% Tween‐20). Biotinylated SUMO‐peptides were immobilized on streptavidin tips. Loaded tips were then immersed in a solution of the TRAF6 MATH domain, which had been diluted to the relevant concentration in BLI buffer. Association data were collected at room temperature at an orbital shake speed of 1,000 rpm (sampling rate) until the signal plateaued. Subsequently, TRAF6 bound tips were transferred to a well containing the above buffer, and dissociation data were collected until the signal plateaued. Due to the fast kinetics of the interaction, we elected to calculate *K*_d_ values using the steady‐state signal of the association step. The raw association data of a SUMO‐only control was subtracted from that of the SUMO‐peptides. The normalized signal of the association step was averaged over 10 seconds after reaching a plateau and plotted against the concentration of TRAF6 MATH domain. The binding curve was fit to Equation (1) in Kaleidagraph^58^ using non‐linear least‐squares fitting to determine the dissociation constant.

*Enrichment sorting of MACS‐presorted library*: To isolate the best TRAF6 binders, we performed a five‐round enrichment sort using the MACS‐sorted library (MACSLib) as the input. On each day, the library was sorted for TRAF6 binding as described above (*Bacterial FACS preparation*) using a single permissive gate set to collect successfully expressed TRAF6 binders. The gate was set manually each day using positive and negative binding controls. Selection for TRAF6 binding was gradually increased by using a lower concentration of T6cc each day (concentrations used: 300 nM, 100 nM, 30 nM, 10 nM, 3 nM). Collected cells were grown overnight before splitting half of the pool to continue the sort and the other half to harvest for plasmid DNA and subsequent Illumina sequencing. We performed two duplicate 5‐day enrichment experiments, generating 10 total pools for deep sequencing.

*Nonbinding clone FACS sorting*. Using the unenriched (pre‐MACS) library as input, a gate was drawn to define the region where peptide‐expressing cells are found in the absence of TRAF6. This gate was used to collect 2 * 10^4^ cells in the presence of a high TRAF6 concentration ([T6cc] = 6 μM) to isolate clones with no detectable binding to TRAF6.

*Illumina amplicon preparation*: Figure S2 gives an overview of this procedure. Sorted pools were grown overnight at 37°C in LB, and bulk plasmid DNA was harvested by QIAprep miniprep kit (Qiagen). We then PCR amplified the variable region of the plasmids from each cell‐sorted pool, appending a MmeI restriction site to the 5′ end. At the 3′ end, we appended: (a) an unused, randomized 9 nt barcode UID sequence, (b) a 6 nt indexing sequence for multiplexing (Illumina TruSeq), and (c) a custom reverse‐read annealing sequence. Barcodes are given in Table S1, amplicon construction is depicted in Figure S2, and a lookup table is provided in Table S2. Amplified fragments were digested with MmeI. A double‐stranded DNA fragment with a 2 nt overhang matching the MmeI cut site was then ligated to each MmeI‐cleaved fragment. This fragment contained the standard 5′ Illumina adapter sequence and one of 24 preselected 5 nt barcodes for sample multiplexing. 5′ and 3′ Illumina anchoring sequences were appended to the amplicons in a subsequent PCR amplification. More than 50 amplicons were Sanger sequenced (QuintaraBio) to assess amplicon quality, which revealed the expected sequences and variable positions. The sequencing length of each amplicon was 65 nt, so forward and reverse paired‐end 40 nt reads overlapped by 15 nt. Immediately prior to Illumina sequencing, the MIT BioMicro Center verified fragment size for all pools by agarose gel and multiplexed all pools at equimolar amounts.

*Illumina data collection and processing*: Illumina sequencing was performed on a NextSeq500. The reads were demultiplexed using custom python scripts: https://github.com/jacksonh1/NGS_demultiplexing. Reads that did not exactly match one of the barcode/index pairs (first 5 nts of the forward read and first 6 nts of the reverse read, respectively) were discarded. Additionally, we required each of the first 5 nts of the forward read to have a Phred score of 20 or greater. Next, the “reformat.sh” tool from the BBTools suite (Version 38.94) was used to de‐interleave the paired‐end reads and filter for reads with an average Phred score greater than or equal to 20 (using the parameter: “minavgquality = 20”).^59^ In our dataset, the forward reads covered the entire variable region of the displayed peptide. Therefore, reverse reads were discarded after de‐interleaving, and only the higher‐quality forward reads were used for further analysis. For each sample, we used custom Python scripts to count the abundance of each sequence in each sample at the DNA level, using an alignment‐based counting strategy. Here, the forward reads were aligned to a counting template sequence covering the variable region of the display construct: *********CCT***GAA*********CCGG, where * represents variable nucleotide positions. Sequences that mismatched 3 or more times to constant positions of the template (non * positions) were removed. Sequence counts were then further collapsed to just the TRAF6 motif region: *********CCT***GAA*********. The result was a list of sequences and their associated read counts for each sample. NGS data, processed data files, and Python scripts are available at https://github.com/jacksonh1/TRAF6_screen and https://doi.org/10.6084/m9.figshare.20485914.v3 (see supplementary information for file descriptions). All sequence logos in this study were generated using the logomaker python library.^60^

*Enrichment data analysis*: For each replicate enrichment experiment, the nucleotide sequences were translated into amino acid sequences, and only those DNA sequences coding for peptides matching the xxxPxExxx motif were kept for further analysis. Amino acid sequences containing the characters “*” or “X” were then removed (corresponding to sequences containing stop codons or “N” bases). Read frequencies were calculated by dividing the read count of each sequence in each sample by the total number of reads in that sample. When a sequence had fewer than 20 reads, the frequency was set to 0 to minimize effects from low read counts. To determine a set of TRAF6 binding sequences, we first removed any sequences that did not have 50 or more reads on at least one of the five enrichment days or the input library (MACSLib). We then filtered for sequences with 20 or more reads on Day 4 and/or Day 5 in either replicate enrichment. The resulting list of binders was then further filtered to include only those sequences that enriched two or more times (defined as an increase in read frequency from one day to the next day) during either enrichment replicate, yielding a final list of 236 unique TRAF6‐binding peptides.

*Nonbinder data analysis*: The NGS data from the nonbinder FACS sample (*Nonbinding clone FACS sorting*) were analyzed to define sequences of peptides that do not bind to TRAF6. Sequences were filtered to include only those DNA sequences coding for peptides matching the xxxPxExxx motif and having a read count of 20 or more. Amino acid sequences containing the characters “*” or “X” were removed. The final list of nonbinders contained 1,200 unique peptides.

*Generation of PSSM for proteome scanning*: To generate a PSSM from the enrichment and nonbinder data, we used pLogo, which uses log‐odds‐based scoring to generate a PSSM from a given set of foreground sequences and background sequences.^35^ We used the 236 unique TRAF6‐binding peptides determined from the enrichment experiment as the foreground and the 1,200 unique nonbinding peptides from the nonbinder sample as the background.

*Scoring motif matches in the proteome*: To generate a table of TRAF6 motif instances in the proteome, we used SLiMSearch 4^36^ to find all matches to the consensus TRAF6 binding motif (regex: “…P.E…[FYWHDE]”) in the human proteome. The PSSM generated with pLogo (described above), was then used to score the hits from SLiMSearch using custom python scripts. We used the SLiMSearch “shared functional annotations” feature to allow filtering the hits to proteins that share Gene Ontology (GO) terms with TRAF6. The set of GO terms used to filter the hits can be restricted by the likelihood that a given term is shared by any two proteins in the proteome (“sig” or “*p*‐value”). We used this feature and custom python code to create filters of different cutoff values (sig < = 0.01, 0.001, 0.0001, and 0.00001) to allow filtering hits to those that share TRAF6 GO terms with sig less than or equal to the given cutoff value. The HIPPIE database was used to label motif instances in proteins that are annotated to interact with TRAF6.^43^ AlphaFold 2 structure predictions for the human proteome were downloaded from the AlphaFold Protein Structure Database.^38^, ^39^, ^40^ Per‐residue AlphaFold pLDDT scores for each motif in the table (+3 flanking residues) were extracted from the predicted structures using custom python scripts. For proteins in the table with no corresponding AlphaFold Protein Structure Database entry, the pLDDT columns were left blank.

*Selection of binding and nonbinding peptides for modeling studies*: 48 binders were chosen from the binding sequences identified in the enrichment experiment for structure‐based modeling (MD binders). Three of the 48 binders (RNVPEESDF, RNVPEESTW, and WNMPAEYDF) came from an earlier analysis of the enrichment data and are not present in the final set of 236 binders. However, all three sequences enrich at least once during the enrichment experiment and are likely real binders despite not making the final cutoff. In addition, 41 nonbinder sequences were selected from the nonbinder pool for structural modeling (MD nonbinders).

*Computational Rosetta modeling*: The pipeline for modeling mutated peptide interactions proceeded as follows. First, all structures were alchemically mutated onto the crystal structure of TRAF6 bound to the CD40* peptide (seq: KQEPQEIDF, PDB: 1LB6) using FoldX. For each mutated pose, we used Rosetta relax to remove steric and angle violations. Next, the Rosetta FlexPepDock module was used to create 500 poses of each using the lowres_preopt flag to more aggressively sample the space in case of necessary residue rearrangement. The talaris2013 score function was used for all model scoring in Rosetta. The top pose by Rosetta score was isolated from each mutated sequence and used to rationalize residue preferences for the binders. The Rosetta version used was rosetta_bin_linux_2017.08.59291_bundle.

*Scoring peptide binding affinity*: We implemented two different computational pipelines for scoring peptide binding to TRAF6: FlexPepBind (FPB)^33^ and an in‐house protocol based on computing a *detaching temperature* (DetachT) by using short molecular dynamics simulations at increasing temperatures.

Structures were prepared using TRAF6‐CD40* complex structure 1LB6 as a model. All sequences were 9 amino acids long and shared the PxExxAr short linear TRAF6‐interacting motif (TIM6). We assumed that all peptides bound in the canonical TRAF6 binding groove with a position similar to that of the CD40* peptide KQEPQEIDF.^19^

Binding energies were computed using the FPB program implemented in Rosetta version 3.6 with scoring function ref2015.^61^ We generated models of peptide‐protein complexes starting with structure 1LB6 (chains A, TRAF6 MATH domain, and B, CD40*), first relaxing the structure with the Rosetta Fast‐Relaxation protocol to remove internal clashes and any angle violations in the receptor and CD40*. We then introduced point mutations into the CD40* peptide, keeping the backbone atoms fixed and optimizing the sidechain conformations of mutated residues using the fixed‐backbone design package with Resfile flag.^62^ Next, the Rosetta FPB module was used to sample 100 variations of the docking pose for each peptide, allowing both backbone and sidechain atoms to move, using the refinement flag, and applying harmonic constraints around the crystallographic distances between the peptide and TRAF6 to reduce the conformational sampling space. Specifically, we restrained backbone hydrogen bond distances between the peptide residue in position (+0) and TRAF6 residue G472 and between the peptide residue in position (+2) and TRAF6 residue G470 (using the observed distances in structure 1LB6). Models were ranked based on total interface score, calculated as the sum over energy terms contributed by interface residues of both partners. Interface residues were defined as those with Cβ (Cα for Gly) within 8 Å of any atom in the TRAF6 protein. We used the lowest‐interface score complex for our analysis. The following is the command‐line flag array for modeling peptides using Rosetta: ($name indicates a wildcard inserted to match the peptide to be run).

$rosdir/FlexPepDocking.static.linuxgccrelease ‐s $name\_Dock_0001.pdb ‐native $nativepdb ‐lowres_preoptimize ‐pep_refine ‐nstruct 500 ‐use_input_sc ‐ex1 ‐ex2 ‐out:file:silent $name\_Dock. silent ‐out:file:silent_struct_type binary.

For the Detach‐T protocol, the crystal structure was initially minimized and equilibrated for 20 ns with CHARMM36a using ACEMD code. The resulting structure was then mutated using the VMD‐Mutator tool to introduce changes into structure 1LB6.^63^ We ran MD simulations with the temperature increasing from 300 up to 1,000 K, using a temperature step of 10 K every 100 ps for a total time of 5 ns, restraining protein CA that were more than 15 Å from the binding pocket to avoid protein diffusion in the unit cell. For each peptide, we recorded the temperature at which the distance between the geometrical center of TRAF6 residue 471 and the center of mass of the peptide segment composed of residues P (0) − x (+1) − E (+2) increased to greater than 7 Å.

*Molecular Dynamics simulation of a subset of TRAF6‐peptide complexes*: For 89 complexes (48 MD binders and 41 MD nonbinders), we performed short molecular dynamics (MD) simulations to identify key interactions or disruptive elements that influence peptide binding. Simulations were performed in NAMD using the CHARMM36m force field.^64^, ^65^ Each of 89 TRAF6‐peptide complexes was solvated with a 15 Å pad of TIP3P water (resulting in a final simulation box of ≈80,000 atoms). Simulations were performed at a constant pressure of 1 atm and temperature of 300 K, a non‐bonded cut‐off of 12 Å, rigid bonds between heavy atoms and hydrogen atoms,^66^ and particle‐mesh Ewald (PME) long‐range electrostatics.^67^ All complexes were first subjected to 1,000 energy minimization steps. Relaxed models were then equilibrated for 50 ns using a time step of 2 fs with all Ca atoms restrained by a 10 kcal mol^−1^ Å^−2^ spring constant. Finally, 100 ns production runs were done using ACEMD,^68^ with non‐bonded cut‐off and PME parameters set as in the equilibration phase, and the time step increased to 4 fs. To prevent protein diffusion in the water box, a restraining spring constant (5 kcal mol^−1^ Å^−2^) was applied to all Cα atoms of the protein more than 15 Å from the peptide‐binding pocket.

Structures from the production runs were analyzed to determine root mean square deviations (RMSD), root mean square fluctuations (RMSF), and the presence/absence of specific interactions (hydrogen bonds, salt‐bridges) using a Donor (D)‐to Acceptor (A) distance cutoff of 3.2 Å; hydrogen bonds were additionally required to have an A–D–H angle of <30°. We also checked for structurally favorable aromatic sidechain arrangements. In particular, cation‐pi interactions were defined using the distance between the indole/phenyl group centroid and the guanidium centroid or amino group for Arg/Lys, respectively, and the angle between the respective planes. The angle was defined between the normal vectors to the planes of the sidechain rings, the guanidium group, or the positively charged groups. To qualify as cation‐pi interaction, the distance had to be below 5.5 A. If the sidechains had an angle between 45 and 135°, the cation‐pi interaction was defined as T‐shaped, otherwise as stacked.^69^, ^70^ We applied a similar definition for pi–pi interaction, setting the distance threshold between the centroids of the two aromatic rings to 7 Å, and the angle range between 75° and 90° for T‐shaped or < 15° for stacked (parallel displaced or vertical).^71^, ^72^, ^73^ MATH domain charge distributions for Figure 6 were computed using the Coulombic electrostatic potential (ESP) tool in ChimeraX.^74^

ACKNOWLEDGEMENTS

We thank the Koch Institute's Robert A. Swanson (1969) Biotechnology Center for technical support, specifically for peptide synthesis and flow cytometry expertise and services. We also thank the MIT Biophysical Instrumentation Facilty for access to instrumentation, and acknowledge use of the MIT Engaging and C3DDB computing clusters.

## AUTHOR CONTRIBUTIONS

**Jackson Clark Halpin:** Conceptualization (supporting); data curation (equal); formal analysis (equal); funding acquisition (supporting); investigation (equal); methodology (equal); project administration (equal); software (lead); validation (equal); visualization (equal); writing – original draft (equal); writing – review and editing (equal). **Dustin Whitney:** Conceptualization (supporting); data curation (supporting); formal analysis (supporting); funding acquisition (supporting); investigation (equal); methodology (equal); project administration (equal); visualization (supporting); writing – original draft (supporting); writing – review and editing (supporting). **Federica Rigoldi:** Conceptualization (supporting); data curation (equal); formal analysis (equal); funding acquisition (supporting); investigation (equal); methodology (equal); project administration (equal); validation (equal); visualization (equal); writing – original draft (equal); writing – review and editing (supporting). **Venkatesh Sivaraman:** Data curation (supporting); methodology (supporting); software (supporting). **Avinoam Singer:** Investigation (supporting); project administration (supporting); validation (supporting); writing – review and editing (supporting). **Amy Keating:** Conceptualization (lead); funding acquisition (lead); project administration (equal); resources (lead); supervision (lead); writing – original draft (equal); writing – review and editing (equal).

## FUNDING INFORMATION

Research reported in this publication was supported by the National Institute of General Medical Sciences of the National Institutes of Health under Awards F32 GM137510 to Jackson C. Halpin, F32 GM114959 to Dustin Whitney and R01 GM129007 to Amy E. Keating, Avinoam Singer received support from National Institute of General Medical Sciences training award T32 GM007287. Jackson C. Halpin was also supported by an award from the Ludwig Center for Molecular Oncology at MIT. Federica Rigoldi was supported by a Progetto Roberto Rocca Post‐doctoral Fellowship. This work was supported in part by the Koch Institute Support (core) Grant P30‐CA14051 from the National Cancer Institute. The content herein is solely the responsibility of the authors and does not represent the official views of any of the funding organizations.

## Supporting information


**Appendix S1** Supporting Information

## Data Availability

The data that support the findings of this study are openly available on Github at https://github.com/jacksonh1/TRAF6_screen and figshare (https://doi.org/10.6084/m9.figshare.20485914.v3).
