# Supplementary material for: Molecular determinants of TRAF6 binding specificity suggest that native interaction partners are not optimized for affinity
Source: Protein Sci. 2022 Oct 26;31(11):e4429. doi: 10.1002/pro.4429 (PMC9597381; doi:10.1002/pro.4429)
Supplement: Supplementary file 1 — Appendix S1 Supporting Information [file PRO-31-e4429-s001.pdf]

Supplementary Information

Halpin et al., *Molecular determinants of TRAF6 binding specificity suggest that native interaction partners are not optimized for affinity*

Extended methods

Stepwise protocol for amplicon generation of Illumina substrates

Sorted pools from the enrichment and nonbinder experiments were grown overnight in 10 mL LB + 25 µg/mL chloramphenicol (OD600 > 1.0) and then plasmid DNA from each pool was isolated (QIAprep miniprep kit using manufacturer's instructions). The resulting DNA pools were subjected to the following procedure (see Figure S3):

1. PCR1 amplified the variable region of the bulk plasmid DNA and attached a 5′ top strand overhang containing 12 nt of flanking DNA and a TCCACC MmeI recognition sequence. MmeI cuts 20 nt 3′ of its recognition site on the top strand and 18 nt 5′ on the bottom strand. The MmeI site appended in PCR1 in our construct was designed such that MmeI digestion results in a bottom strand 3′ overhang of 'AG' to match the overhang of our DNA adapters. If other adapter is chosen, make sure to generate the appropriate overhang. The top strand 3′ primer appends both a 9 nt unique identifier (UID) and a 6 nt index sequence. The appended UIDs in our analysis were not used in this work. After 5 cycles at T_a = 60 °C, the next 20 cycles were run at T_a = 66 °C such that only full-length fragments were reproduced. Phusion High-Fidelity Polymerase in HF Buffer was used (0.5 µL Phusion/50 µL reaction) for all PCR steps. The 6 nt index sequences that we used are indicated in Table S1. PCR products were purified with the Zymo DNA Clean and Concentrate Kit (Genesee).

   PCR Recipe:
   10 µL Phusion HF Buffer 5X
   1 µL dNTPs (10 mM each dNTP)
   1.1 µL PCR1a fwd primer (10 µM)
   1 µL PCR1a rev primer (10 µM)
   10 µL Ligated DNA template
   1 µL Phusion Polymerase
   <u>25 µL water</u>
   50 µ total

   Thermal Cycler:
   1. 3 min 98 °C
   2. 30 sec 98 °C
   3. 30 sec 60/66 °C*
   4. 30 sec 72 °C
   5. Repeat steps 2–4 14X
   6. 3 min 72 °C
   7. Hold 4 °C

   *: After 5 cycles with T_a = 60 °C, add 1.5 µL PCR1b reverse primer (10 µM) and resume with T_a = 66 °C

2. MmeI (NEB) was used as in the NEB instructions to cleave PCR1 products. The resulting DNA fragments should have a ~200 ng PCR1 product, and are used as input DNA for each digestion in the following recipe:

   Digestion Recipe:
   9 µL PCR1 product
   2 µL CutSmart 10X buffer
   1 µL SAM
   6 µL water
   <u>2 µL MmeI (4U)</u>
   20 µL total

   Thermal Cycler:
   1. 60 min 37 °C
   2. 20 min 80 °C (heat deactivation)
   3. Hold 4 °C

T4 DNA Ligase (NEB) was used to anneal adapters (Table S1) assigned by pool identity. The adapters, which contain both the 5′ Illumina forward sequencing primer sequence and a 5′ 5-nt barcode, each having a 'TC' top-strand 3' overhang for annealing to the designed 'AG' bottom-strand 3′ overhang left by MmeI cleavage of purified PCR1 products. Ligated products were then run on a 1% Agarose gel containing 10 µL GelGreen/100 mL total gel volume and bands ~117 nt were excised and purified with the Zymoclean Gel DNA Recovery Kit. Purified products were eluted in 20 µL water.

**Ligation Recipe:**	**Thermal Cycler:**	
15 µL MmeI-digested DNA	1. 30 min	25 °C
2 µL T4 DNA Ligase Buffer 10X	2. 10 min	65 °C (heat deactivation)
2 µL DNA Adapter (6 µM)	3. Hold	4 °C
1 µL T4 DNA Ligase (400 U)		
20 µL total		

3. PCR2 amplifies the purified ligated amplicons to add the 5′ Illumina anchor sequences to each strand of the amplicon. PCR2 products were purified with the Zymo DNA Clean and Concentrator Kit, and eluted in 20 µL water. Selected samples were then sent for Sanger sequencing (QuintaraBio) to monitor amplicon quality and appropriate barcoding.

**PCR Recipe:**	**Thermal Cycler:**	
10 µL Phusion HF Buffer 5X	1. 3 min	98 °C
1 µL dNTPs (10 mM each dNTP)	2. 30 sec	98 °C
3.1.µL Fwd primer (10 µM)	3. 30 sec	66 °C
3.2.µL Rev primer (10 µM)	4. 30 sec	72 °C
10 µL Ligated DNA template	5. Repeat steps 2–4	14X
1 µL Phusion Polymerase	6. 3 min	72 °C
25 µL water	7. Hold	4 °C
50 µL total		

Description of proteome motif table (Table S4) filters and scores

TRAF6-binding SLiMs must be accessible for binding. The hits in our table can be filtered by IUPred score [1] to only include hits that are predicted to be disordered (*e.g.* IUPred score > 0.4). However, IUPred score is not a guarantee of accessibility. The AlphaFold pLDDT score is reported to be a good predictor of disorder [2], so we included the average and maximum AlphaFold pLDDT scores of the motif (+/- 3 flanking residues) within the predicted structure of the protein [2–4]. For the average score, we recommend a cutoff of < 65 but caution that this will likely remove some instances where a motif is still accessible, despite the high pLDDT score. The maximum pLDDT score of any residue within the motif +/- 3 residues on each side is also reported, as this may detect cases in which most of the motif is disordered but proximity to a folded domain structure limits accessibility. For this filter, we recommend setting it to consider hits with a maximum pLDDT score of less than 70.

Proteins involved in similar biological processes as TRAF6 are more promising candidate interaction partners [5]. To identify MATH domain binding motifs in proteins that share functions in common with TRAF6, we used Gene Ontology (GO) annotations [6,7]. Specifically, we used SLiMSearch to retrieve GO terms for TRAF6, where each term has an associated p-value representing the likelihood that any 2 proteins in the proteome share that term by chance (p-value from SLiMSearch [5]). The table of proteome hits can then be filtered to include proteins that share 1 or more TRAF6 GO terms with a p-value below a given threshold. Smaller p-value cutoffs result in more general GO terms being removed from the list of terms used in the filter and provide greater stringency. We provide several different p-values as options and suggest p=0.01 as a starting point.

Many proteins have been reported to interact with TRAF6 without identification of the mode of interaction. We used the HIPPIE database [8] to identify which of the proteome windows that we evaluated are in proteins that are already annotated to be TRAF6 interaction partners. A high-scoring motif match in a protein annotated as a TRAF6 interaction partner could indicate that its interaction with TRAF6 is likely to occur through the MATH domain.

Our structural analysis identified several sequence features that disfavor or prevent PxE peptides from binding to the MATH domain (Table 2). We added filters to the table to identify candidate motifs having unfavorable residues, such as a proline at positions (+1) to (+5), a large/medium residue (QHILFYW) at position (+3), or a positively charged residue (RK) at positions (+3), (+4), or (+5).

Supplementary Figures

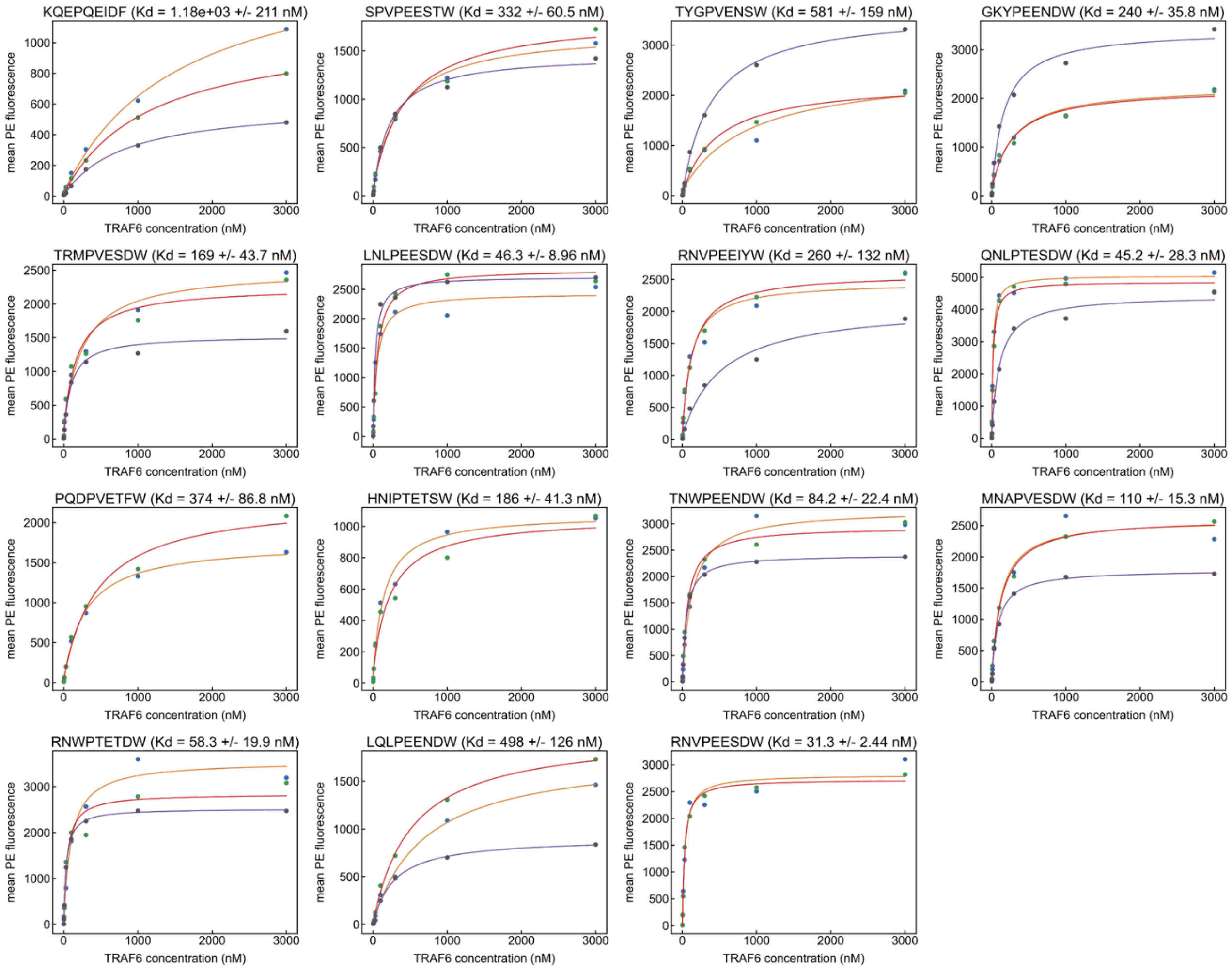

Figure S1. Binding curves from single-clone FACS titrations. Mean PE fluorescence is plotted against TRAF6 concentration and fit to a standard binding equation (equation 1). The mean K_d^* from fitting each replicate independently and the associated standard error of the mean is shown above each graph.

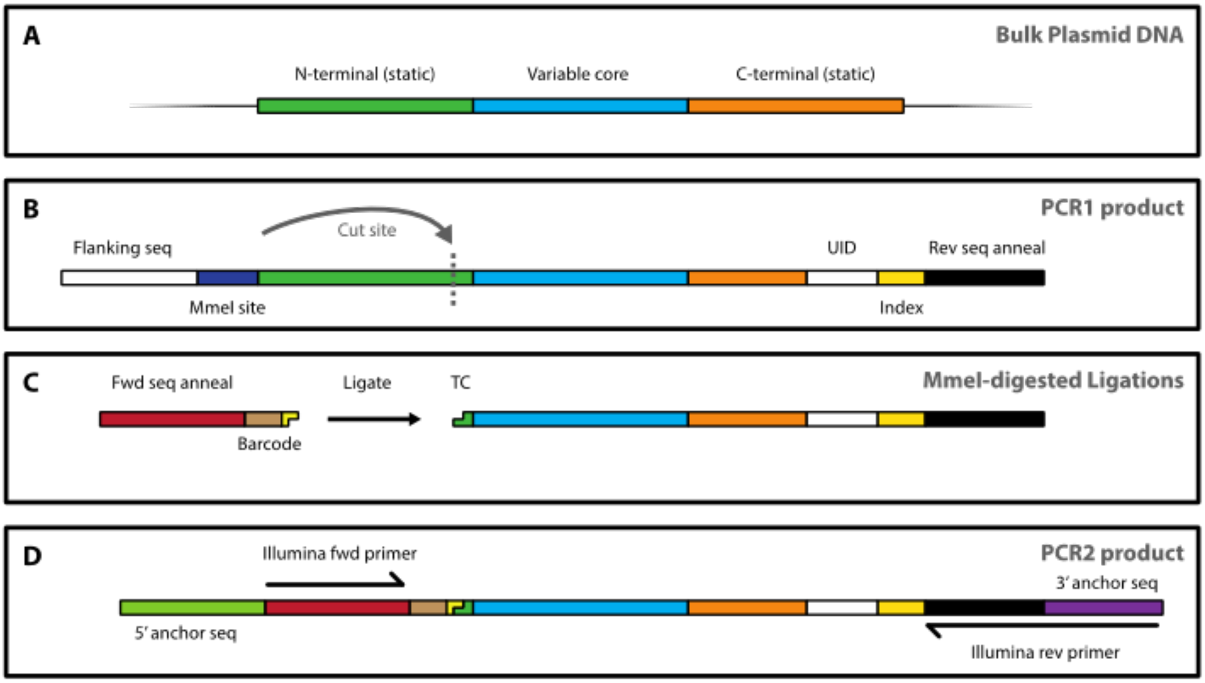

Figure S2. Schematic of amplicon preparation from sorted plasmid pools.

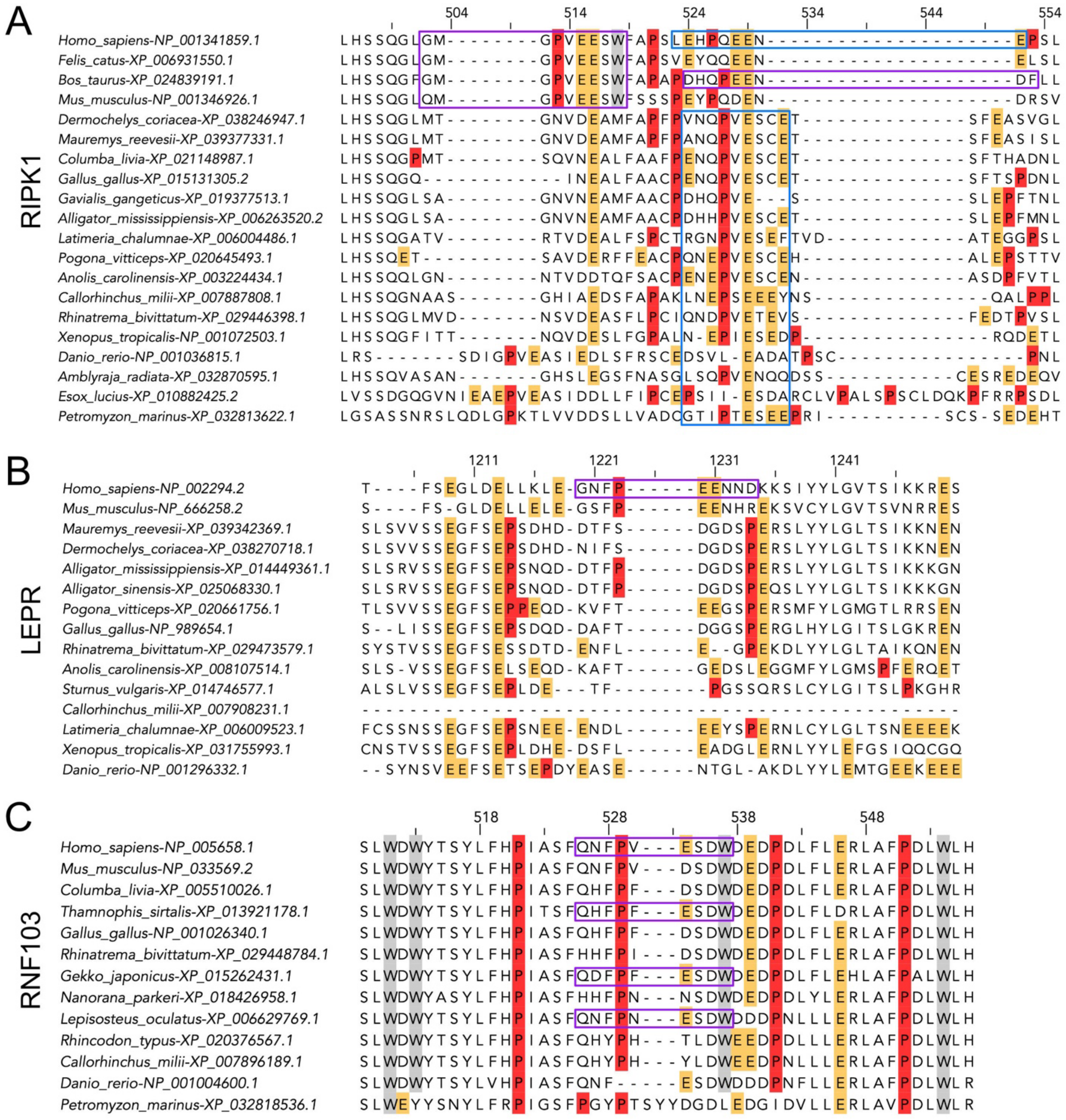

Figure S3. Sequence conservation of selected TRAF6 motifs. For RIPK1 (A), LEPR (B), and RNF103 (C), homologous proteins were retrieved using homologene [9]. Orthologs from a representative set of vertebrates were selected and their sequences were aligned using Clustal-Omega [10]. Multiple sequence alignment images were generated using Jalview [11]. Motifs with sequence features that our model predicts are favorable for high-affinity binding to TRAF6 are indicated in purple boxes. For RIPK1, putative TIM6 motifs that contain Glu rather than Trp at (+5) are indicated in the blue box.

List of supplementary data files deposited to public repositories*

*available at https://github.com/jacksonh1/TRAF6_screen and https://doi.org/10.6084/m9.figshare.20485914.v3

Supplementary data file	Contents	column names	column description
enrichment_rep1_readcounts.csv	merged read counts for enrichment replicate 1	pre-enrichment (MACSlib)	read counts in MACSlib (enrichment input)
		day_1	read counts after round 1
		day_2	read counts after round 2
		day_3	read counts after round 3
		day_4	read counts after round 4
		day_5	read counts after round 5
		seq	nucleotide sequence
enrichment_rep2_readcounts.csv	merged read counts for enrichment replicate 2	pre-enrichment (MACSlib)	read counts in MACSlib (enrichment input)
		day_1	read counts after round 1
		day_2	read counts after round 2
		day_3	read counts after round 3
		day_4	read counts after round 4
		day_5	read counts after round 5
		seq	nucleotide sequence
MD_sequences.xlsx	sequences used in structural modeling studies	Binders	48 MD binder peptide sequences
		Nonbinders	41 MD nonbinder peptide sequences
enrichment_rep1_readcounts-processed.csv	enrichment replicate 1 - read counts after filtering*	seq	nucleotide sequence
		AA_seq	amino acid sequence
		pre-enrichment (MACSlib)	read counts in MACSlib
		day_1	read counts after round 1
		day_2	read counts after round 2
		day_3	read counts after round 3
		day_4	read counts after round 4
		day_5	read counts after round 5
enrichment_rep2_readcounts-processed.csv	enrichment replicate 2 - read counts after filtering*	seq	nucleotide sequence
		AA_seq	amino acid sequence
		pre-enrichment (MACSlib)	read counts in MACSlib
		day_1	read counts after round 1
		day_2	read counts after round 2
		day_3	read counts after round 3
		day_4	read counts after round 4
		day_5	read counts after round 5
nonbinder_readcounts-processed.csv	nonbinder pool - read counts after filtering* final 1200 nonbinders	seq	nucleotide sequence
		AA_seq	amino acid sequence
		read counts	read counts
final_binder_list.txt	final set of 236 binder sequences	N/A	amino acid sequence
Supplementary_Table_S4.xlsx	TRAF6 motifs in the human proteome with PSSM score	columns explained at the top of the file	N/A

*Filtering: Nucleotide sequences were collapsed to just the motif (*********CCT***GAA*********) and then translated into amino acid sequence. Peptides which did not match the motif (xxxPxExxx) or which contained a "*" or "X" character were removed. Any sequences that didn't have a read count of 20 or more in at least one of the read count columns were removed.

Supplementary tables

Table S1. TRAF6 and peptide display constructs and index/barcode sequences used for multiplexing NGS samples.

**TRAF6 MATH-plus-coiled-coil construct - T6cc**
MA – (BAP tag) – GGSS – HHHHHH – GSGSGSM – (human TRAF6 residues 310-504)
ATGGCTGGAGGCCTGAACGATATTTTCGAAGCTCAGAAAATCGAATGGCACGAGGACACTGGTGGCTCGAGCCACCA<br>TCACCATCACCATGGTTCGGGCAGCGGATCGATGGATCATCAGATTCGTGAACTGACCGCGAAAATGGAAACCCAGAG<br>CATGTACGTGAGCGAACTGAAACGCACGATTCGTACACTGGAAGATAAAGTGGCGGAAATTGAAGCGCAGCAGTGCA<br>ATGGCATCTATATATGGAAAATTGGCAATTTTGGCATGCATCTGAAGTGCCAGGAAGAAGAAAAACCGGTGGTGATTC<br>ATAGCCCGGGATTCTATACGGGCAAACCGGGTTACAAACTGTGCATGCGTCTGCATCTTCAACTGCCGACCGCGCAGC<br>GTTGCGCCAATTACATCAGCCTGTTTGTGCATACCATGCAGGGCGAATATGATAGCCATCTGCCGTGGCCGTTCCAAGG<br>CACCATTCGTCTGACCATTTTAGATCAGAGCGAAGCGCCGGTGCGTCAGAATCATGAAGAAATTATGGATGCGAAACC<br>GGAACTGCTGGCTTTTCAACGTCCTACCATTCCGCGTAATCCGAAAGGCTTCGGCTATGTGACGTTTATGCACCTGGAA<br>GCTCTGCGCCAGCGTACCTTTATCAAAGATGATACCCTGCTTGTGCGGTGTGAAGTGAGCACCCGCTTTGATTAA
**TRAF6 monomer construct - T6m**
(human TRAF6 residues 350-501) – LE – HHHHHH
ATGAATGGCATCTATATATGGAAAATTGGCAATTTTGGCATGCATCTGAAGTGCCAGGAAGAAGAAAAACCGGTGGT<br>GATTCATAGCCCGGGATTCTATACGGGCAAACCGGGTTACAAACTGTGCATGCGTCTGCATCTTCAACTGCCGACCGC<br>GCAGCGTTGCGCCAATTACATCAGCCTGTTTGTGCATACCATGCAGGGCGAATATGATAGCCATCTGCCGTGGCCGTTC<br>CAAGGCACCATTCGTCTGACCATTTTAGATCAGAGCGAAGCGCCGGTGCGTCAGAATCATGAAGAAATTATGGATGCG<br>AAACCGGAACTGCTGGCTTTTCAACGTCCTACCATTCCGCGTAATCCGAAAGGCTTCGGCTATGTGACGTTTATGCACC<br>TGGAAGCTCTGCGCCAGCGTACCTTTATCAAAGATGATACCCTGCTTGTGCGGTGTGAAGTGAGCACCCTAGAACACC<br>ATCACCATCACCACTAA
**Peptides tested for cell-surface binding were displayed in an eCPX fusion construct with the following sequence:**
(eCPX residues 1 - 183 from ref. [12]) –<br>MKKIACLSALAAVLAFTAGTSVAGGQSGQSGDYNKNQYYGITAGPAYRINDWASIYGVVGVGYGKFQTTEYPTYKHDTSDY<br>GFSYGAGLQFNPMENVALDFSYEQSRIRSVDVGTWILSVGYRFGSKSRRATSTVTGGYAQSDAQGQMNKMGGFNLKYRY<br>EEDNSPLGVIGSFTYTEKSRTAS–GGGSGGGSDYKDDDDKGGGSGGGSGSGGQSGRGS–PTNKAPHP-xxxPxExxx-<br>PDDLPGSNT
ATGAAAAAAATTGCATGTCTTTCAGCACTGGCCGCAGTTCTGGCTTTCACCGCAGGTACTTCCGTAGCTGGAGGGCAGT<br>CTGGGCAGTCTGGTGACTACAACAAAAACCAGTACTACGGCATCACTGCTGGTCCGGCTTACCGCATTAACGACTGGG<br>CAAGCATCTACGGTGTAGTGGGTGTGGGTTATGGTAAATTCCAGACCACTGAATACCCGACCTACAAACACGACACCA<br>GCGACTACGGTTTCTCCTACGGTGCGGGTCTGCAGTTCAACCCGATGGAAAACGTTGCTCTGGACTTCTCTTACGAGCA<br>GAGCCGTATTCGTAGCGTTGACGTAGGCACCTGGATTTTGTCTGTTGGTTACCGCTTCGGGAGTAAATCGCGTCGCGC<br>GACTTCTACTGTAACTGGCGGTTACGCACAGAGCGACGCTCAGGGCCAAATGAACAAAATGGGCGGTTTCAACCTGAA<br>ATACCGCTATGAAGAAGACAACAGCCCGCTGGGTGTGATCGGTTCTTTCACTTACACCGAGAAAAGCCGTACTGCAAG<br>CGGAGGAGGCAGTGGTGGTGGGAGCGATTACAAAGATGACGATGACAAAGGCGGCGGTAGTGGGGGAGGCAGTGG<br>TAGCGGCGGCCAGTCTGGCCGTGGTTCTCCCACTAATAAGGCCCCGCATCCCAAACAAGAACCTCAGGAAATCGATTT<br>CCCGGACGATCTGCCGGGTAGCAACACATGATAA
**SUMO peptide construct used in BLI:**
MAG – (BAP tag) – DTGGSS – (TEV cleavage site) – HHHHHHH – GSGSG – (SUMO) – GSGSGGSGSG – xxxPxExxx
**Index and barcode sequences used to multiplex NGS samples**
Index sequences: ATCACG, CGATGT, TTAGGC, TGACCA, ACAGTG, GCCAAT, CAGATC, ACTTGA, GATCAG
Barcode sequences: ACTCG, ACTGT, AATGC, AGTCA, ATACG, ATAGC, CGATC, CTAAG, CTCGA, CGAAT, CTGGT, CGGTT, GACTT, GTTCA, GATAC, GAGCA, GATGA, GTCTG, TCGGA, TGACC, TACTG, TCCAG, TCGAC, TAGCT

Table S2. fastq file sample key

barcode file name	Sample
barcode_0	enrichment - replicate 1 - day 1
barcode_1	enrichment - replicate 1 - day 2
barcode_2	enrichment - replicate 1 - day 3
barcode_3	enrichment - replicate 1 - day 4
barcode_4	enrichment - replicate 1 - day 5
barcode_6	enrichment - replicate 2 - day 1
barcode_7	enrichment - replicate 2 - day 2
barcode_8	enrichment - replicate 2 - day 3
barcode_9	enrichment - replicate 2 - day 4
barcode_10	enrichment - replicate 2 - day 5
barcode_20	MACSLib pool*
barcode_5	nonbinder pool

*used as input for enrichment experiments

Table S3. Features of candidate TRAF6 interaction motifs and experimentally verified motifs. All columns are from SLiMsearch [5] except for "normalized pLogo PSSM score", "AF2 pLDDT", "number of favorable residues" and "verified or predicted TRAF6 motif". "AF2 pLDDT" is the average AlphaFold pLDDT score of the motif (+/- 3 flanking residues) within the predicted structure of the protein [2–4]. "Number of favorable residues" is the number of residues in the motif that are predicted to favor TRAF6 binding, defined as: N at (-2), E at (+1), D at (+4), and W at (+5).

Accession	Name	sequence	normalized pLogo PSSM score	IUPred	AF2 pLDDT	number of favorable residues	Verified or Candidate TRAF6 motif
O43187	IRAK2	SNTPEETDD	0.555	0.75	43.10	3	Verified
P25942	CD40	KQEPQEINF	0.082	0.764	44.85	0	Verified
P51617	IRAK1	PPSPQENSY	0.094	0.517	35.85	0	Verified
P51617	IRAK1	PNQPVESDE	0.469	0.687	34.68	2	Verified
P51617	IRAK1	RQGPEESDE	0.391	0.863	42.17	2	Verified
Q7Z434	MAVS	CHGPEENEY	0.279	0.557	53.23	1	Verified
Q8IUC6	TICAM1	CQEPEEMSW	0.591	0.736	38.02	2	Verified
Q96CG3	TIFA	SSSPTEMDE	0.198	0.789	62.55	1	Verified
Q9Y616	IRAK3	PSIPVEDDE	0.208	0.54	42.41	1	Verified
O00237	RNF103	QNFPVESDW	0.850	0.163	38.71	3	Candidate
Q13546	RIPK1	GMGPVEESW	0.455	0.606	43.48	1	Candidate
P48357	LEPR	GNFPEENND	0.487	0.31	32.49	2	Candidate

References

[1] B. Mészáros, G. Erdős, Z. Dosztányi, IUPred2A: context-dependent prediction of protein disorder as a function of redox state and protein binding, Nucleic Acids Research. 46 (2018) W329–W337. https://doi.org/10.1093/nar/gky384.

[2] K. Tunyasuvunakool, J. Adler, Z. Wu, T. Green, M. Zielinski, A. Žídek, A. Bridgland, A. Cowie, C. Meyer, A. Laydon, S. Velankar, G.J. Kleywegt, A. Bateman, R. Evans, A. Pritzel, M. Figurnov, O. Ronneberger, R. Bates, S.A.A. Kohl, A. Potapenko, A.J. Ballard, B. Romera-Paredes, S. Nikolov, R. Jain, E. Clancy, D. Reiman, S. Petersen, A.W. Senior, K. Kavukcuoglu, E. Birney, P. Kohli, J. Jumper, D. Hassabis, Highly accurate protein structure prediction for the human proteome, Nature. 596 (2021) 590–596. https://doi.org/10.1038/s41586-021-03828-1.

[3] J. Jumper, R. Evans, A. Pritzel, T. Green, M. Figurnov, O. Ronneberger, K. Tunyasuvunakool, R. Bates, A. Žídek, A. Potapenko, A. Bridgland, C. Meyer, S.A.A. Kohl, A.J. Ballard, A. Cowie, B. Romera-Paredes, S. Nikolov, R. Jain, J. Adler, T. Back, S. Petersen, D. Reiman, E. Clancy, M. Zielinski, M. Steinegger, M. Pacholska, T. Berghammer, S. Bodenstein, D. Silver, O. Vinyals, A.W. Senior, K. Kavukcuoglu, P. Kohli, D. Hassabis, Highly accurate protein structure prediction with AlphaFold, Nature. 596 (2021) 583–589. https://doi.org/10.1038/s41586-021-03819-2.

[4] M. Varadi, S. Anyango, M. Deshpande, S. Nair, C. Natassia, G. Yordanova, D. Yuan, O. Stroe, G. Wood, A. Laydon, A. Žídek, T. Green, K. Tunyasuvunakool, S. Petersen, J. Jumper, E. Clancy, R. Green, A. Vora, M. Lutfi, M. Figurnov, A. Cowie, N. Hobbs, P. Kohli, G. Kleywegt, E. Birney, D. Hassabis, S. Velankar, AlphaFold Protein Structure Database: massively expanding the structural coverage of protein-sequence space with high-accuracy models, Nucleic Acids Research. 50 (2022) D439–D444. https://doi.org/10.1093/nar/gkab1061.

[5] I. Krystkowiak, N.E. Davey, SLiMSearch: a framework for proteome-wide discovery and annotation of functional modules in intrinsically disordered regions, Nucleic Acids Research. 45 (2017) W464–W469. https://doi.org/10.1093/nar/gkx238.

[6] M. Ashburner, C.A. Ball, J.A. Blake, D. Botstein, H. Butler, J.M. Cherry, A.P. Davis, K. Dolinski, S.S. Dwight, J.T. Eppig, M.A. Harris, D.P. Hill, L. Issel-Tarver, A. Kasarskis, S. Lewis, J.C. Matese, J.E. Richardson, M. Ringwald, G.M. Rubin, G. Sherlock, Gene Ontology: tool for the unification of biology, Nature Genetics. 25 (2000) 25–29. https://doi.org/10.1038/75556.

[7] The Gene Ontology Consortium, The Gene Ontology resource: enriching a GOld mine, Nucleic Acids Research. 49 (2021) D325–D334. https://doi.org/10.1093/nar/gkaa1113.

[8] G. Alanis-Lobato, M.A. Andrade-Navarro, M.H. Schaefer, HIPPIE v2.0: enhancing meaningfulness and reliability of protein–protein interaction networks, Nucleic Acids Res. 45 (2017) D408–D414. https://doi.org/10.1093/nar/gkw985.

[9] Database resources of the National Center for Biotechnology Information., Nucleic Acids Res. 44 (2016) D7-19. https://doi.org/10.1093/nar/gkv1290.

[10] F. Sievers, A. Wilm, D. Dineen, T.J. Gibson, K. Karplus, W. Li, R. Lopez, H. McWilliam, M. Remmert, J. Söding, J.D. Thompson, D.G. Higgins, Fast, scalable generation of high-quality protein multiple sequence alignments using Clustal Omega., Mol Syst Biol. 7 (2011) 539. https://doi.org/10.1038/msb.2011.75.

[11] A.M. Waterhouse, J.B. Procter, D.M.A. Martin, M. Clamp, G.J. Barton, Jalview Version 2--a multiple sequence alignment editor and analysis workbench., Bioinformatics. 25 (2009) 1189–1191. https://doi.org/10.1093/bioinformatics/btp033.

[12] J.J. Rice, A. Schohn, P.H. Bessette, K.T. Boulware, P.S. Daugherty, Bacterial display using circularly permuted outer membrane protein OmpX yields high affinity peptide ligands., Protein Sci. 15 (2006) 825–836. https://doi.org/10.1110/ps.051897806.
